# Fingerprinting molecular and isotopic biosignatures on different hydrothermal scenarios of Iceland, an acidic and sulfur-rich Mars analog

**DOI:** 10.1038/s41598-020-78240-2

**Published:** 2020-12-03

**Authors:** Laura Sánchez-García, Daniel Carrizo, Antonio Molina, Victoria Muñoz-Iglesias, María Ángeles Lezcano, Maite Fernández-Sampedro, Victor Parro, Olga Prieto-Ballesteros

**Affiliations:** Centro de Astrobiología (CSIC-INTA), Carretera de Ajalvir km 4, Madrid, Spain

**Keywords:** Biogeochemistry, Environmental microbiology, Planetary science

## Abstract

Detecting signs of potential extant/extinct life on Mars is challenging because the presence of organics on that planet is expected to be very low and most likely linked to radiation-protected refugia and/or preservative strategies (e.g., organo-mineral complexes). With scarcity of organics, accounting for biomineralization and potential relationships between biomarkers, mineralogy, and geochemistry is key in the search for extraterrestrial life. Here we explored microbial fingerprints and their associated mineralogy in Icelandic hydrothermal systems analog to Mars (i.e., high sulfur content, or amorphous silica), to identify potentially habitable locations on that planet. The mineralogical assemblage of four hydrothermal substrates (hot springs biofilms, mud pots, and steaming and inactive fumaroles) was analyzed concerning the distribution of biomarkers. Molecular and isotopic composition of lipids revealed quantitative and compositional differences apparently impacted by surface geothermal alteration and environmental factors. pH and water showed an influence (i.e., greatest biomass in circumneutral settings with highest supply and turnover of water), whereas temperature conditioned the mineralogy that supported specific microbial metabolisms related with sulfur. Raman spectra suggested the possible coexistence of abiotic and biomediated sources of minerals (i.e., sulfur or hematite). These findings may help to interpret future Raman or GC–MS signals in forthcoming Martian missions.

## Introduction

The search for organic compounds is one of the main goals in planetary exploration. Because of its proximity to Earth, location within the habitability zone, and rock similarities with certain regions on Earth, Mars has been one of the key targets for searching for extraterrestrial life. It is thought that liquid water could have once made certain Martian areas inhabitable (e.g.,^1,2^). In that case, simple life, such as bacteria or archaea, might have existed on the red planet and their remnants may still be present underneath the planet’s frozen surface. Because of the complexity of accessing Mars environments and the limitations of accomplishing *in-situ* analysis there, the search for an extant or extinct subsurface biosphere on Mars has largely benefited from the use of extreme environments on Earth with analogies to Mars, as (partial) model systems for gathering certain information for future and ongoing Martian missions.

Amongst the extreme terrestrial environments with a resemblance to Mars, geothermal hot springs and the associated sinter precipitates are well known habitable environments on the early Earth (e.g.,^3,4^ and references therein) that are considered potential analogs of hydrothermal processes on Mars (e.g.,^5–7^). These environments, including impact-generated hydrothermal systems^8,9^, provide a localized source of heat, water, and the necessary materials to harbor life (e.g.,^7,10^). A first step in the search for possible biosignatures on extraterrestrial hydrothermal systems is assessing the presence and state of organic matter. On Mars, the detection at Gale Crater of preserved organic molecules (i.e. thiophenic, aromatic and aliphatic compounds) by the SAM instrument onboard the MSL Curiosity^11,12^ fulfilled that first requirement. Then, investigating the occurrence and distribution of molecular biomarkers in terrestrial hydrothermal systems may give an insight into the fingerprints to seek in similar geothermal environments on Mars (i.e., former mud pots, fumaroles, geysers, or chimneys). Knowing what are the dominant microbial communities in these settings and how they distribute according to physicochemical variables (e.g., temperature, pH, or redox conditions) is essential for interpreting possible biosignatures on analog sites on Mars.

Many mineral assemblages on Mars consisting of clays, sulfates, iron oxides, and hydrated silica have been identified through orbital and land-based observations. Some of these sites show geological and chemical correlation with hydrothermal alteration. Nili Patera, Elysium Planitia, Apollinaris Patera, as well as Gusev, Gale, and Jezero Craters are few examples of potential hydrothermal alteration associated with volcanic edifices (e.g.,^7,10,13^). Gas–water–rock interactions were likely produced in fumaroles, mud pots, and hot springs by volatile sulfur^14^, altering the iron-rich basaltic Martian crust^15^. In the search for Martian life evidences, Jezero crater (landing site of the NASA’s Perseverance rover in 2021) and Gusev crater (landing site of the NASA’s Mars Exploration Rover Spirit in 2004) are particularly interesting targets due to the presence in both of amorphous silica, a mineral phase with high potential to preserve biosignatures on Earth that has been orbitaly identified in Jezero by the Compact Reconnaissance Imaging Spectrometer for Mars (CRISM)^16^ and in situ measured in Gusev by the Spirit rover^17^.

Iceland hydrothermal sites (e.g., Krýsuvík, Hveragerdi, or Námafjall), resulting from circulating water by magma plumes heating and formed in contact with high-Fe basalts^18^, are considered a natural laboratory for analog studies of Mars^19^, containing hot springs, geysers, mud pots, and steam vents^20^ with abundant opaline silica deposits and elemental sulfur as in Gusev Crater^17^. Astrobiological features on Iceland include the aforementioned hot springs and their associated microbial populations, including extremophilic life combining polar and hydrothermal systems. Space-related missions on Iceland go back to some 50 years, when NASA astronauts trained for the Apollo 11 moon landing on Icelandic soil^21^. Now, various projects develop on this remote “Land of Fire and Ice” to understand habitability and environmental-microbial interactions with an eye on future astrobiological missions to Mars. Ecological studies have used DNA sequencing^22,23^ or lipid biomarkers analysis^24,25^ to determine the composition and community structure of hot springs microbial mats from geochemically diverse Icelandic geothermal fields, assessing in some cases the effect of physicochemical variables such as sulfide concentration, pH or temperature^22^. The discovery of hydrothermal deposits on Mars has also raised the interest in hydrothermal siliceous deposits on Iceland due to their capacity to preserve microbial biosignatures, with an eye on preparing future mission targeting potential biosignatures in analogous deposits on Mars^26^. While the search for microbial biosignatures on Icelandic hydrothermal deposits have focused on hot springs, other hydrothermal settings with higher relevance for the presently inactive hydrothermal Mars (e.g., fossilized fumaroles or mud pots) remain unexplored.

**Figure 1:**
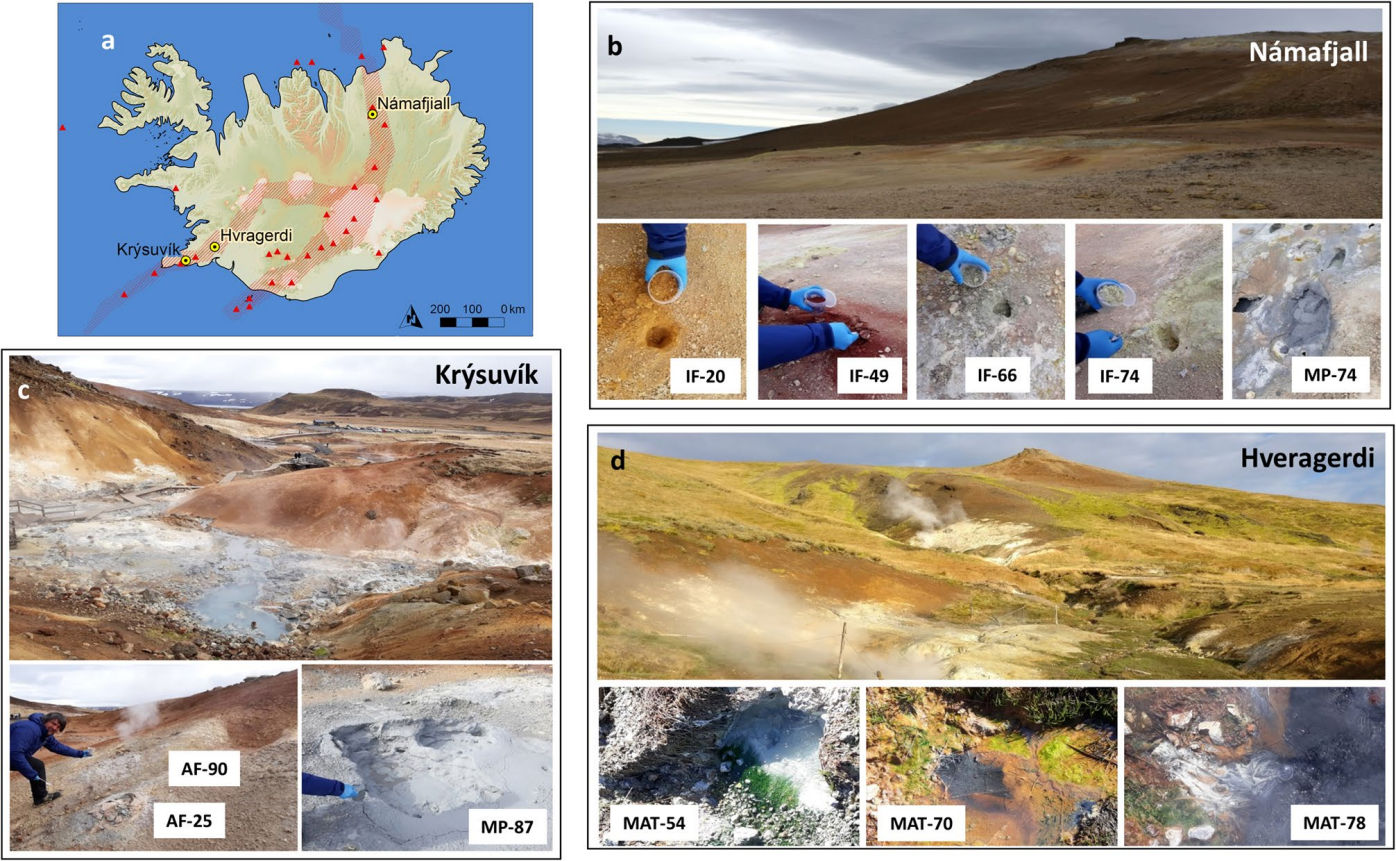
Icelandic geothermal areas investigated in this work (**a**) throughout the Mid-Atlantic Ridge (red dashed area with red triangles representing volcanoes) traversing the island; (**b**) Námafjall, (**c**) Krýsuvík, and (**d**) Hvragerdi. The map in (**a**) was built with ArcGIS Desktop 10.8 (https://desktop.arcgis.com/). Hydrothermal samples include four inactive fumaroles (IF) and one mud pot (MP) in Námafjall; two active fumaroles (AF) and one MP in Krýsuvík, and three hot spring biofilms (MAT) in Hveragerdi. The numbers in the sample names indicate the discrete temperature recorded in situ at the time of collection.

Here we wanted to move forward to explore the microbial fingerprint in geothermal substrates others than hot springs, with different mineral sequence and progressively lower availability of liquid water to simulate different scenarios with a potential analogy to hydrothermal Mars. We investigated three different geothermal fields along the Mid-Atlantic Ridge: Krýsuvik, located on the Reykjanes peninsula; Hveragerdi, to the southeast of the Hengill volcanic complex; and Námafjall, adjacent to Krafla volcano in northern Iceland (Fig. 1, see Methods section). These currently active hydrothermal areas show fluid-dominated mud pots and gas-driven fumaroles that, in combination with the meteoric cold water, produced a wide range of alteration products, like sulfates, sulfides, amorphous silica phases, clay minerals, and Fe–Ti oxides^18,20^. We explored the molecular and isotopic composition of lipid biomarkers in four hydrothermal substrates (hot springs, mud pots, active and inactive fumaroles). A total of 11 samples were analyzed for interpreting their biological signature and carbon metabolism (Fig. 1); three hot spring biofilms at 54 °C (MAT-54), 70 °C (MAT-70), and 78 °C (MAT-78), in Hveragerdi; two mud pots at 74 °C (MP-74), in Námafjall, and 87 °C (MP-87), in Krýsuvik; and soil samples from two active fumaroles at 25 °C (AF-25) and 90 °C (AF-90), in Krýsuvik, and four inactive fumaroles at 20 °C (IF-20), 49 °C (IF-49), 66 °C (IF-66), and 74 °C (IF-74), in Námafjall. All samples showed pH values ranging from 1 to 6 (Table 1). The diversity and distribution of biomarkers in each regime were analyzed in relation to their mineralogy and variation of different physicochemical variables (temperature, pH, water, or light accessibility) to understand their influence in determining quantitative or qualitative patterns. By fingerprinting the molecular and isotopic distribution patterns of lipid biomarkers in different hydrothermal scenarios, we aim at gathering as much knowledge as possible to constrain better (i) where to search for hypothetical biosignatures of past or present life on Mars and (ii) how to interpret them.

**Table 1:** Location and description of the 11 hydrothermal samples in the four Icelandic regimes; hot spring biofilms (MATs), mud pots (MPs), active fumaroles (AFs), and inactive fumaroles (IFs).

Site	Sample	Latitude	Longitude	Temp. °C	pH	Water (%)^a^	System description	Sample description
Hveragerdi	MAT-54	64° 01′205	21° 23′657	54	6	93	Hot spring pot	Dark green mat at the pot side
Hveragerdi	MAT-70	64° 01′204	21° 23′690	70	6	98	Hot spring (boiling)	Orange-pale green mat at river side
Hveragerdi	MAT-78	66° 01′204	21° 23′690	78	6	96	Hot spring (boiling)	Whitish fibers at river side
Námafjall	MP-74	63° 53′359	16° 48′594	74	2	82	Dark grey mud pot	Dark grey mud
Krýsuvík	MP-87	63° 53′712	22° 03′316	87	2	52	Pale grey mud pot	Pale grey mud
Krýsuvík	AF-25	63° 53′709	22° 03′303	25	2	57	Cooler fumarole	White-pale yellow solid material
Krýsuvík	AF-90	63° 53′708	22° 03′302	90	1	71	Steaming fumarole	White-pale yellow solid material
Námafjall	IF-20	63° 38′392	16° 48′696	20	3	0.04	Non-steaming fumarole	Ochre soil substrate
Námafjall	IF-49	63° 38′392	16° 48′695	49	3	0.02	Non-steaming fumarole	Vermilion soil substrate
Námafjall	IF-66	63° 38′392	16° 48′697	66	4	0.03	Non-steaming fumarole	Grey soil substrate
Námafjall	IF-74	63° 38′392	16° 48′694	74	1	0.02	Non-steaming fumarole	Pale yellow soil substrate

^a^Content of interstitial water gravimetrically determined by subtracting from the initial sample weight the constant mass reached after drying the sample at 50 °C in an oven for 48–72 h.

## Results and discussion

### Molecular and isotopic biosignatures in three Icelandic hot spring biofilms

**Figure 2:**
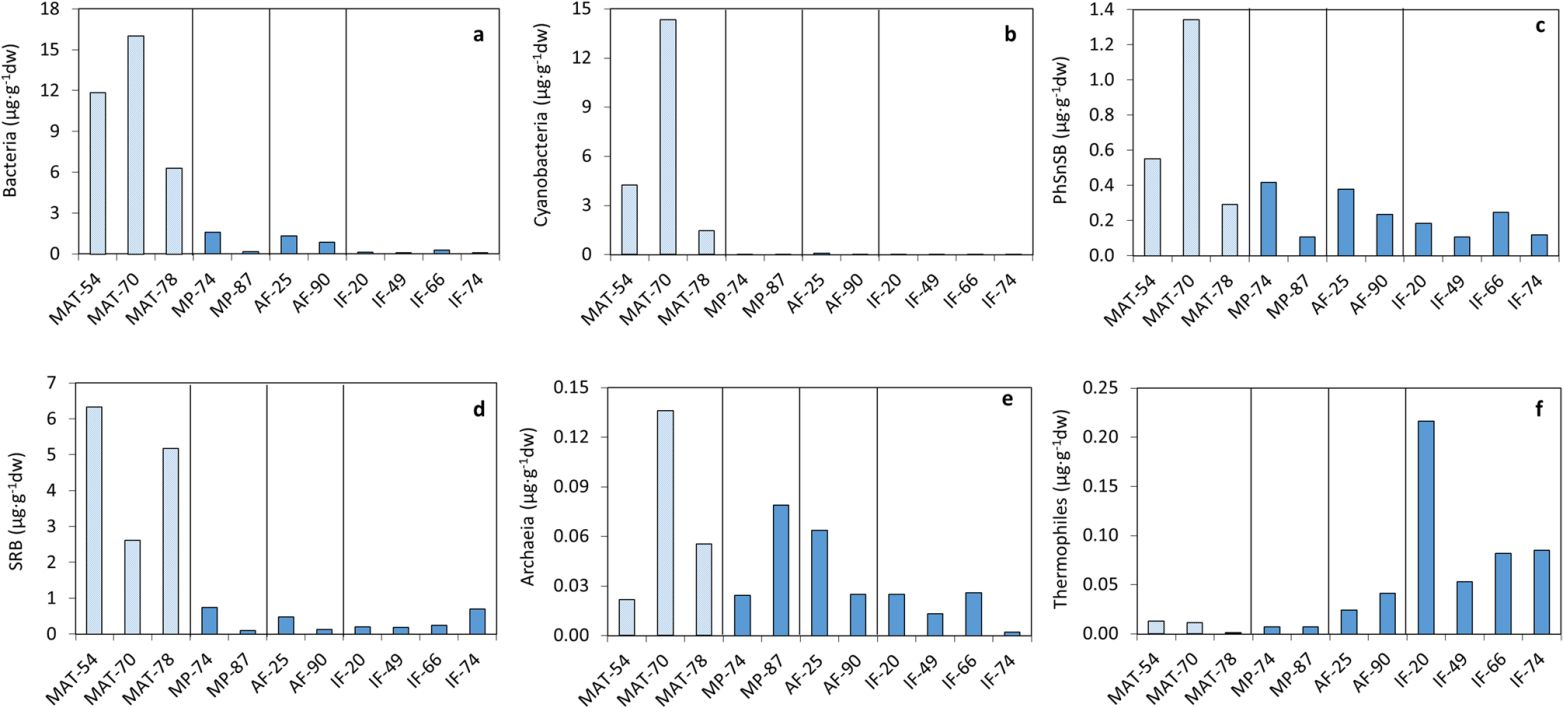
Relative contribution of microbial input sources to the samples biomass in the four hydrothermal regimes, inferred from the presence (µg·g^−1^ of dry weight) of lipid biomarkers of (**a**) bacteria (sum of *n*-fatty acids from 16:0 to 18:0;^58^); (**b**) cyanobacteria (sum of *n*-heptadecane, isomeric *n*-heptadecenes, and monomethylalkanes of C_17_, C_18_ and C_19_^59–62^; diploptene^63^; and 16:1ω7, 18:2ω6, and 18:3ω6 fatty acids^62,64,65^); (**c**) photosynthetic sulfur and non-sulfur bacteria or PhSnSB (sum of the *n*-alkanols C_16_, C_17_, and C_18_^66^); (**d**) sulfate-reducing bacteria or SRB (sum of phytane^67^; *i/a*-pairs of 15:0, 17:0, and 15:1 fatty acids^35^; and 16:1ω5, 17:1, and 18:1ω5 fatty acids^35,68,69^); (**e**) archaea (squalane^70,71^); and (**f**) thermophiles (sum of dicarboxylic acids;^72^). See Text S1 for details on the uses and limitations of the approach.

The abundance and distribution of lipid biomarkers represent a mixture of contributions from all organisms present and past (when remnants are well preserved) in an environment^27^, providing insight on the overall community structure and biogeochemical processes occurring at a location (Text S1–S2). In the Hveragerdi hot springs, different lipid molecular patterns (Fig. S1) revealed contributions from diverse biological sources (see Text S3–S4). In MAT-54 and MAT-70, the relative abundance and isotopic composition of lipids (hydrocarbons, fatty acids, and hopanoids) diagnostic of cyanobacteria and other phototrophs (Text S1–S2) indicated a dominance of these microorganisms in their microbial community. This was consistent with previous descriptions of microbial mats in Hveragerdi hot springs collected from waters from 44 to 66 °C^24^, where the thermophilic cyanobacterium *Mastigocladus* was dominant. Contribution from other phototrophs was deduced from the abundance (mostly in MAT-70) of lipid biomarkers of photosynthetic sulfur (*Chlorobiaceae*) and non-sulfur (*Chloroflexi*) bacteria (Fig. 2). Although specific biomarkers of *Chloroflexi* such as hentriacontatriene (C_31:3_) or HMW wax esters^25^ were not detected in this study, the presence of these green non-sulfur bacteria was considered likely due to different reasons. First, previous investigations on Hveragerdi reported the abundance of the *Chloroflexus* genus in different hot spring mats^24,25^, where it coexisted with cyanobacteria (mostly *Mastigocladus*), surrounding it for protection against sulfur and high temperatures, known to inhibit the cyanobacterium growth^24,28^. Second, the macroscopic aspect of our MAT-70 (Fig. 1d) coincided largely with that of *Chloroflexus-*rich biofilms (i.e., green-orange gelatinous and filamentous biomass) described by others^24,25^. Third, the slightly enriched compound-specific and (mostly) bulk δ^13^C ratios observed in the three biofilms (Fig. S2) relative to δ^13^C signatures purely cyanobacterial (Text S2) (see next).

**Figure 3:**
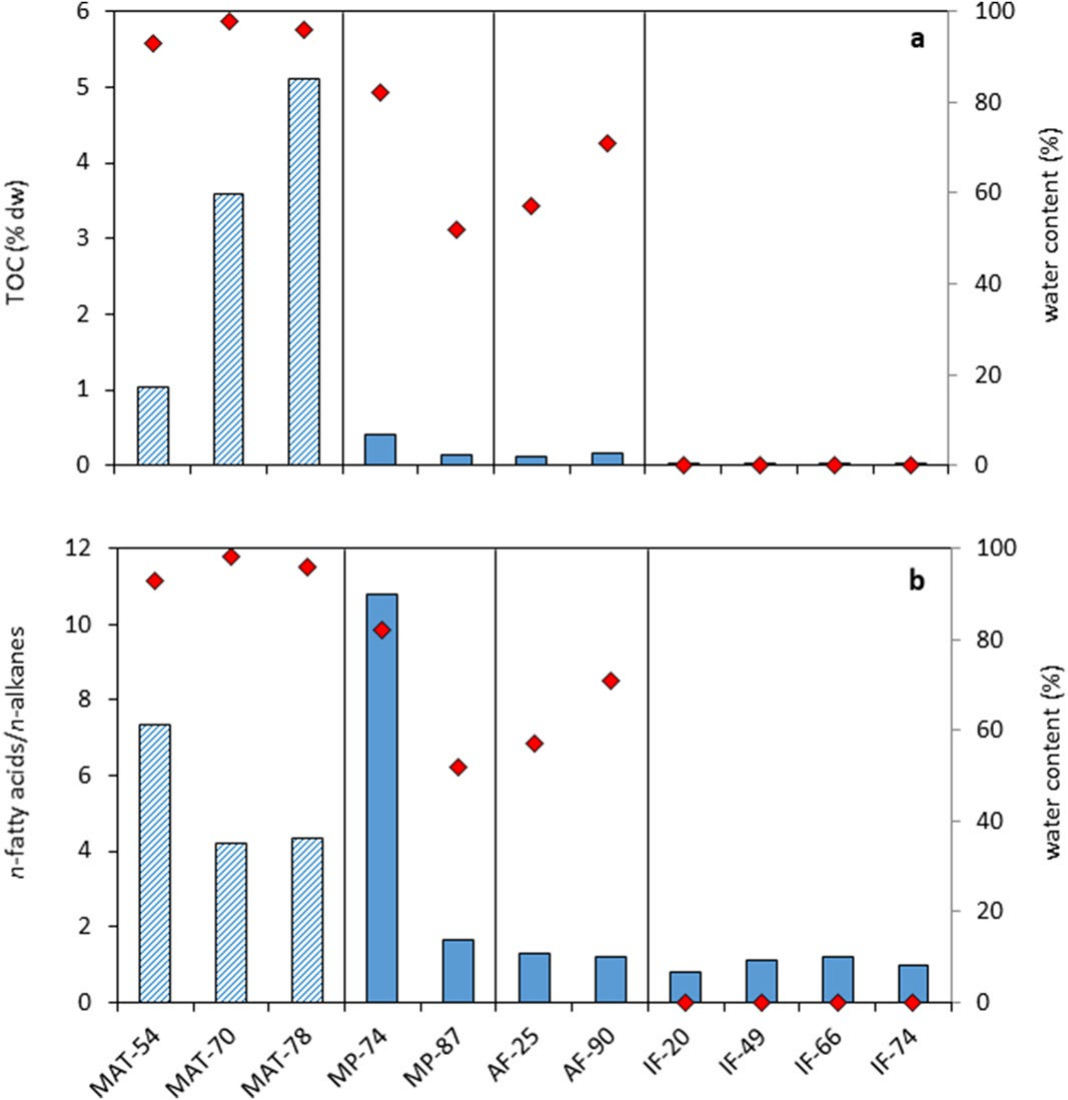
Estimate of (**a**) biomass and (**b**) biological activity degree (blue bars) relative to the sample water content (red diamonds) in the four hydrothermal substrates; hot spring biofilms (MATs), mud pots (MPs), active (AFs) and inactive (IFs) fumaroles. Total biomass is accounted for as the content of total organic carbon (TOC, % of dry weight). The extent of freshness or biological activity was assessed based on the relative abundance of functional groups (i.e. carboxylic acids) over saturated straight-chain (*normal*) alkanes (i.e. the *n*-fatty acids/*n*-alkanes ratio) (see Text S1).

The bulk and molecular δ^13^C composition of MAT-54 and MAT-70 suggested that autotrophic carbon fixation in the two biofilms was mostly conducted through two major pathways (Text S4), consistent to a microbial community structure dominated by cyanobacteria (i.e., Calvin cycle) and *Chloroflexus* (i.e., 3-hydroxypropionate or 3HP bicycle). On the one hand, bulk δ^13^C in MAT-54 (− 18.2‰) and MAT-70 (− 17.9‰) (Table 2) denoted a slight depletion in ^13^C relative to pure *Chloroflexus* mats (− 14.9‰) growing autotrophically (i.e., through the 3HP bicycle) in similar hot springs from Yellowstone National Park^29^. On the other hand, the wide range of values of compound-specific δ^13^C in both biofilms (i.e., from − 18 to − 30‰; Fig. S2) covered values of carbon acquisition typical of users of the Calvin cycle (from − 19 to − 30‰^30^ and the 3HP bicycle (from − 4 to − 15‰^31^).

**Table 2:** Organic geochemical composition of the studied hydrothermal samples on Iceland.

Sample	TOC (% dw)^a^	TN (% dw)^b^	δ^13^C (‰)^c^	δ^15^Nv(‰)^d^	Total lipids (µg·g^−1^ dw)^e^	*n*-fatty acids/*n*-alkanes^f^	br-C_17_/n-C_17_^g^
MAT-54	1.03	0.18	− 18.2	0.59	27	7	0.069
MAT-70	3.59	0.45	− 17.9	− 0.87	105	4	0.28
MAT-78	5.12	0.88	− 20.8	− 5.4	17	4	0.11
MP-74	0.41	0.09	− 16.1	− 2.7	3.3	11	0.098
MP-87	0.14	nd	− 21.2	nd	0.62	1.6	2.4
AF-25	0.11	nd	− 16.7	nd	3.9	1.3	0.11
AF-90	0.16	nd	− 8.1	nd	2.2	1.2	1.4
IF-20	0.02	nd	− 21.1	nd	1.2	0.80	0.60
IF-49	0.02	nd	− 20.2	nd	0.88	1.1	0.10
IF-66	0.03	nd	− 10.0	nd	1.5	1.2	0.44
IF-74	0.01	nd	− 20.1	nd	1.3	0.99	0.14

^a^Total organic carbon (TOC).

^b^Total nitrogen (TN).

^c^Bulk stable carbon isotopic composition of TOC.

^d^Bulk stable nitrogen isotopic composition of TN.

^e^Total sum of lipids including compounds from the apolar (*n*-alkanes, isomeric *n*-alkenes, isoprenoids), acid (*n*-fatty acids, i/a-fatty acids, mono- and polyunsaturated fatty acids, and cyclopropyl fatty acids), and polar (*n*-alkanols, phytol, and sterols) fractions (see Fig. S1, S3, S4 for details on the lipids distributions).

^f^Ratio of saturated straight-chain (*normal*) fatty acids (*n*-fatty acids) over *n*-alkanes. Estimate of freshness or extent of biological activity.

^g^Sum of branched heptadecanes over *n*-heptadecane. Estimate of the relative abundance of heterotrophs^57^.

In MAT-78, in contrast, the presence of cyanobacteria and other phototrophs was considered a minority (Fig. 2), according to the distribution of lipid biomarkers observed in the sample (Fig. S1). The lower proportion of phototrophs was consistent with the higher temperature in this compared to the other two biofilms, which was over the upper-temperature limit of chlorophyll at ~ 73 °C^32^. The microbial community in MAT-78 was instead dominated by biomarkers of sulfate-reducing bacteria or SRB (e.g., *Firmicutes or δ-Proteobacteria*) (see Text S3). According to others^22,23^, the main primary producers in Icelandic hot springs at temperatures > 66 °C include representatives of the bacterial divisions *Aquificales*, *Deinococcus-Thermus* group, *Thermodesulfobacterium* group, *Thermotogales*, and *Nitrospira* group. In addition, some *Proteobacteria* (γ and β classes) and *Firmicutes* (*Bacilli*) have also been reported in microbial mats from hot springs at 70 ± 4 °C in Hveragerdi^22,23^. The relevant role of sulfur in this sample was supported by the aspect of MAT-78, where grey sulfur-rich deposits covered most of the mat filaments (Fig. 1d). The conditions in this site may be favorable for aerobic sulfur- and hydrogen-oxidizing bacteria on top of the white sulfur-rich mat and for anaerobic sulfur and sulfate reducers in the dark grey undermass (Fig. 1d), as described in similar biofilms from Hveragerdi hot springs^22^.

According to this, the stable carbon isotopic composition of MAT-78 denoted meaningful participation of the reductive acetyl-CoA pathway for the autotrophic incorporation of carbon, with an additional potential contribution from the rTCA pathway (see Text S4). Overall, δ^13^C values in MAT-78 were generally more depleted in ^13^C than those in the other biofilms (Fig. S2), both the bulk (i.e., − 20.8‰) and compound-specific ratios (from − 19.1 to − 34.3‰). Carbon fixation pathways involving a large fractionation of ^13^C that result in depleted δ^13^C signatures (i.e*.*, more negative δ^13^C) may be either the Calvin cycle or the reductive acetyl-CoA pathway (see Text S2). As the presence of phototrophs in MAT-78 was regarded as a minority due to its high temperature, the reductive acetyl-CoA pathway was considered the most plausible pathway to explain the relatively more negative values of δ^13^C in this biofilm, in detriment of the Calvin cycle. Microbial groups using the reductive acetyl-CoA pathway (see Text S2) that has been described in high-temperature hot spring mats in Hveragerdi are *Firmicutes* or δ-*Proteobacteria*^23^. The presence of these microorganisms in MAT-78 would be consistent with the relatively higher abundance of *iso*/*anteiso* fatty acids diagnostic of gram-positive bacteria^33^ and SRB^34,35^ in this relative to the other two biofilms (Fig. S1). In addition, a certain contribution from other autotrophic carbon fixation pathways causing less fractionation than the reductive acetyl-CoA pathway (Text S2), such as the reductive tricarboxylic acid (rTCA), was also considered to explain the relative enrichment of the bulk δ^13^C relative to the lipids-specific δ^13^C values (Fig. S2). *Aquificales* or *Nitrospira* are some of the thermophile microorganisms described in Hveragerdi hot springs^23^ that may fix carbon using the rTCA pathway^36^, a metabolism producing bulk δ^13^C values from − 12 to − 21‰.^30^

### Molecular and isotopic biosignatures in other Icelandic hydrothermal substrates; mud pots and fumaroles

The microbial diversity in hot spring mats has been previously assessed on Iceland, based on DNA^22,23^ and lipid biomarkers^24,25^, whereas that of other hydrothermal substrates with astrobiological relevance such as sulfur-rich and pyroclastic soils remain unexplored. Here, we investigated the molecular and isotopic composition of lipid biomarkers in samples of active geothermal regimes such as mud pots, active and inactive fumaroles to assess biological fingerprints in scenarios with analogies to Mars (e.g. Gusev Crater).

The observed molecular distribution patterns in the mud pots (Fig. S3) and fumaroles (Fig. S4–S5) differed largely from those in the three hot spring biofilms (Fig. S1), revealing a relatively higher contribution of archaea and thermophiles in the former, in detriment of cyanobacteria and other phototrophs (see Text S5). Among regimes, the proportion of bacteria, archaea, SRB, and photosynthetic sulfur and non-sulfur bacteria (PhSnSB) was generally higher in the MPs and AFs, whereas thermophiles were relatively more abundant in the active and (mostly) inactive fumaroles (Fig. 2). The signal of heterotrophs (i.e., br-C_17_/*n*-C_17_ ratio; see Text S1) was greatest in MP-87 and AF-90 (Table 2), which could be related to the presence of heterotrophic members of *Nitrospira*, *Crenarchaeota*, or *Thermodesulfobacterium*, as well as fermentative bacteria (*Thermotogales*), described in Hveragerdi (i.e.,^22^).

Wide ranges of stable carbon isotopic ratios in the mud pot (Fig. S6) and fumarole (Fig. S7) samples supported the contribution of the mentioned different biosources, with the participation of various autotrophic carbon fixation pathways involving large and small fractionations of ^13^C (see Text S6). In the mud pots, primary production appeared to be largely represented by the reductive acetyl-CoA pathway (mostly in MP-87), and a lower proportion of the Calvin cycle (mostly in MP-74). Carbon fixation through these pathways could be attributed to microorganisms previously described on Iceland hydrothermal regimes^23^, such as members of γ- or β-Proteobacteria, in the case of the Calvin cycle^36^, or to members of *Euryarchaeota* (methanogenesis), *Archaeoglobales* (sulfate reduction) or δ-*Proteobacteria* (sulfate reduction) in the case of the reductive acetyl-CoA pathway^36^. Additional contributions from the rTCA or 3HP pathways were also considered to explain the relatively enriched bulk δ^13^C in MP-87 (− 21.2‰) and mostly MP-74 (i.e., − 16.1‰). These alternative routes could have been conducted by thermophilic *Aquificales* (rTCA) and, only in the case of the cooler MP-74, by PhSnSB (i.e., *Chlorobiaceae* and *Chloroflexi*) respectively using the rTCA and 3HP pathways (Text S6). Similar participation of mixed carbon fixation pathways was deduced in the fumarolic substrates, with the reductive acetyl-CoA pathway (archaea and/or SRB) appearing to be dominant in the active systems, and the rTCA pathway playing a relevant role in the inactive fumaroles, likely due to the greatest presence of thermophiles (e.g., *Aquificales*, *Nitrospira,* or *Chlorobiaceae*) in this regime (Fig. 2f).

The compositional differences between the Icelandic mud pots and fumaroles relative to the hot spring biofilms was illustrated by the distribution of samples in three groups (i.e., MAT-54 and MAT-78; MAT-70; and rest of samples) in a diagram of Principal Components Analysis (PCA) (Text S8). While the samples showing the lowest biomass (i.e., mud pots and fumaroles) clustered together and showed a negative relationship with TOC, the three hot spring biofilms with relatively higher biomass plotted oppositely in the PCA diagram and grouped in two different clusters based on their different microbial composition (Fig. S8). Such differences were related to the abundance of either SRB (MAT-54 and MAT-78), or cyanobacteria, PhSnSB, and archaea (MAT-70) (Fig. 2). In contrast, mud pots and fumaroles were rather associated with thermophiles and δ^13^C. In terms of metabolism, samples AF-90 and IF-66 showed similarities (Fig. S8) likely associated to a relatively higher incorporation of inorganic carbon through the 3HP and/or TCA pathways (Text S7).

### Influence of environmental variables on the biological fingerprint on Icelandic geothermal environments analog to Mars

The biological fingerprint in the Icelandic geothermal substrates was analyzed in relation to a number of environmental variables (water content, pH, and temperature) to understand their influence on the microbial composition (see Text S8 for details).

**Figure 4:**
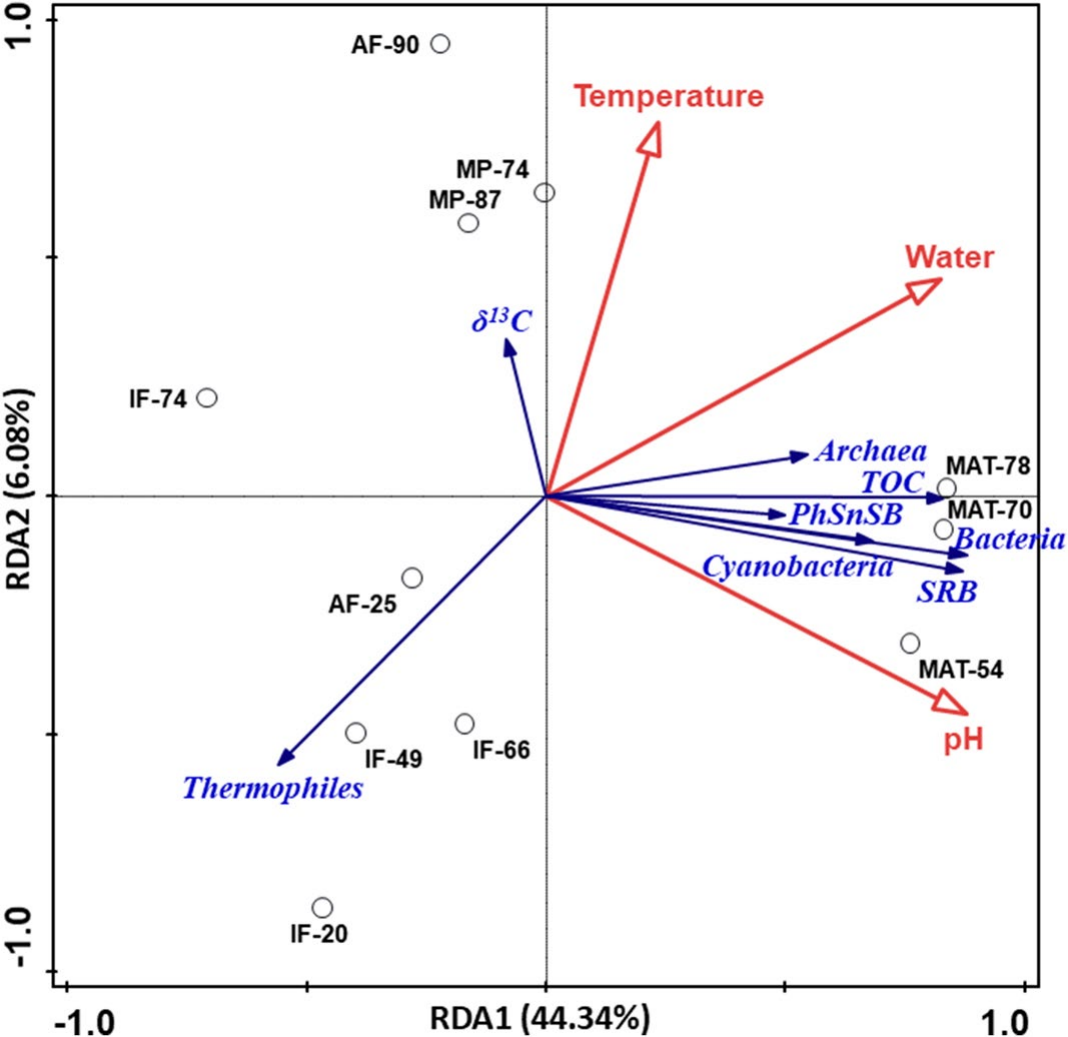
Ordination plot of Redundancy Analysis (RDA) on the Icelandic hydrothermal samples. The blue vectors represent the nine compositional variables tested (see Fig. S8) and the red vectors represent the environmental variables selected for assessing the influence on the organic composition; pH, water, and temperature. See Fig. 1 for description on the samples naming. The axis RDA1 and RDA2 explained together 50.42% of the compositional variability of the samples.

Water and pH resulted the variables influencing most the total amount of biomass and its microbial composition in the Icelandic hydrothermal samples according to a Redundancy Analysis (RDA) (Fig. 4). Both environmental variables showed positive correlation with TOC and the microbial biomarkers of bacteria, cyanobacteria, SRB, PhSnSB, and archaea (Fig. 4). The contribution of microorganisms sensitive to low pH values such as cyanobacteria^37^ to the total biomass was essential in the three biofilms, mostly MAT-70 (Fig. 2b). In the range of values considered here (i.e*.,* from 1 to 6), the greatest biomass was thus produced in settings with circumneutral pH and highest supply and turnover of water (i.e., continuously renewed running water; Text S8).

In contrast to water and pH, temperature did not seem such a determining variable in most of the organic variables attending to the RDA (Text S8). Only thermophiles showed a negative correlation with temperature, and plotted together with the low-temperature fumaroles (Fig. 4). Still, despite the low relationship between temperature and most of the biomarkers, a few patterns could be identified. For instance, the signals of bacteria, SRB, or PhSnSB were generally stronger at temperatures of 66–70 °C (Fig. 2). For cyanobacteria, the general variation with temperature could not be detected, as the growth of these microorganisms was anyway negligible in other substrates than biofilms due to their low pH^37^. Still, in the samples where cyanobacteria were well detected (i.e., the three biofilms; Fig. 2b), their abundance decreased considerably at temperatures above 70 °C^32^. Interestingly, the strongest signal of thermophile biomarkers was found in one of the virtually dry samples (inactive fumaroles) with the lowest temperature (i.e., IF-20; Fig. 2f). This apparent anomaly was hypothesized to be related to the characteristic episodic nature of thermal activity in hydrothermal systems such as fumaroles (see text S8), where the continuous variability of temperature with time^38,39^ allows the alternation in time of microbial communities with different tolerance to temperature^40^. The temporal variation of microbial populations with temperature imprints the substrate with biosignatures of both present and past communities^39,41^. Thus, the highest detection of thermophile biomarkers in IF-20 was considered to be likely recording a larger presence of thermophiles in the past, when a much higher temperature than that measured during the study sampling surely took place.

In sum, water and pH were the environmental factors affecting most (i) the total biomass (quantitative level), and (ii) the microbial community structure (qualitative level) in the Icelandic hydrothermal substrates, whereas temperature appeared to influence only the distribution of thermophiles.

### Interpreting the biological fingerprint of the Icelandic hydrothermal regimes in a mineralogical context

**Figure 5:**
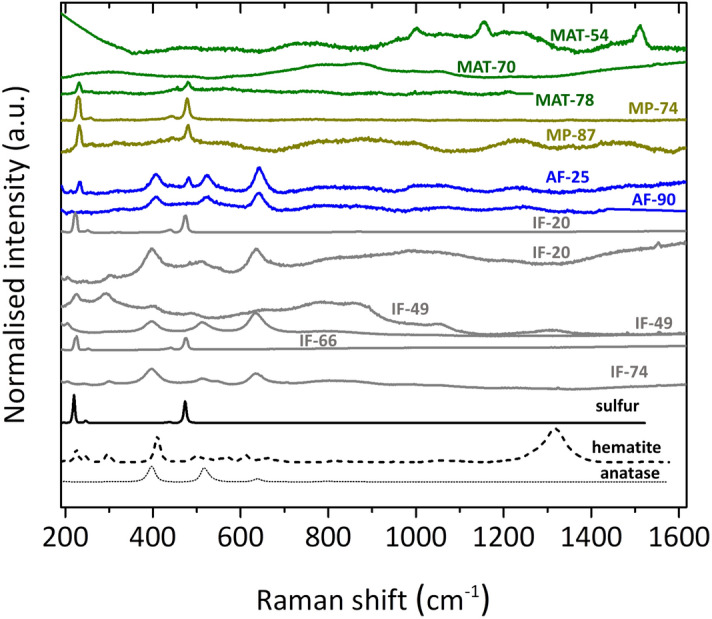
Raman spectra of the minerals identified in the Icelandic hydrothermal regimes; hot spring biofilms (MATs; green), mud pots (MPs; brown), active fumaroles (AFs; blue), and inactive fumaroles (IFs; grey). At the bottom, Raman spectra of minerals from the RRUFF database are shown in black for comparison and initial mineral identification. The spectrum of MAT-54 corresponds to carotenoids, according to Gall et al.^73^. Different Raman spectra profiles depending on the spot measured in IF-20 and IF-49 are caused by mineral heterogeneities.

The mineralogy of the hydrothermal substrates incorporating the biomarkers was also characterized. Overall, minerals containing sulfur and titanium oxide were measured by XRD and Raman to be ubiquitous in the samples (Table 3 and Fig. 5), while other minerals (hematite, pyrite, natroalunite, or clays) were only present in specific samples (Text S9). The presence of amorphous silica suggested by XRD curve profiles in some samples (Table 3) was supported by Fourier Transform Infrared (FTIR) spectroscopy in the near infrared (Text S9; Fig. S9). Sample alteration minerals are function of the local temperature (Fig. S10), redox conditions, and fluid acidity that permits mobilization of ions at acidic hot conditions (see Fig. S10). Mineralogy characterization revealed that phases rich in sulfur (pyrite or elemental sulfur) or titanium (anatase) dominated at samples with temperatures ≥ 66 °C. In contrast, other alteration phases such as hematite, sulfates (natroalunite), clays (kaolinite, montmorillonite), or zeolites (heulandite) were progressively more abundant as hydrothermal activity and temperature decreased (Table 3; Fig. 5).

**Table 3:** Mineral composition of the eleven Icelandic hydrothermal samples (biofilms or MATs, mud pots or MPs, active fumaroles or AFs, and inactive fumaroles or IFs), with presence/absence notation, based on X-Ray Diffraction and Fourier Transform Infrared Spectroscopy.

Sample	Anatase	Sulfur	Pyrite	Quartz	Hematite	Natroalunite	Kaolinite	Montmorillonite	Heulandite	Amorphous silica
MAT-54								+	+	
MAT-70										
MAT-78		+	+							
MP-74	+	+	+				+			+
MP-87		+	+				+			
AF-25	+					+	+			+
AF-90	+	+								+
IF-20	+			+						
IF-49	+				+					
IF-66		+								+
IF-74	+	+								+

**Figure 6:**
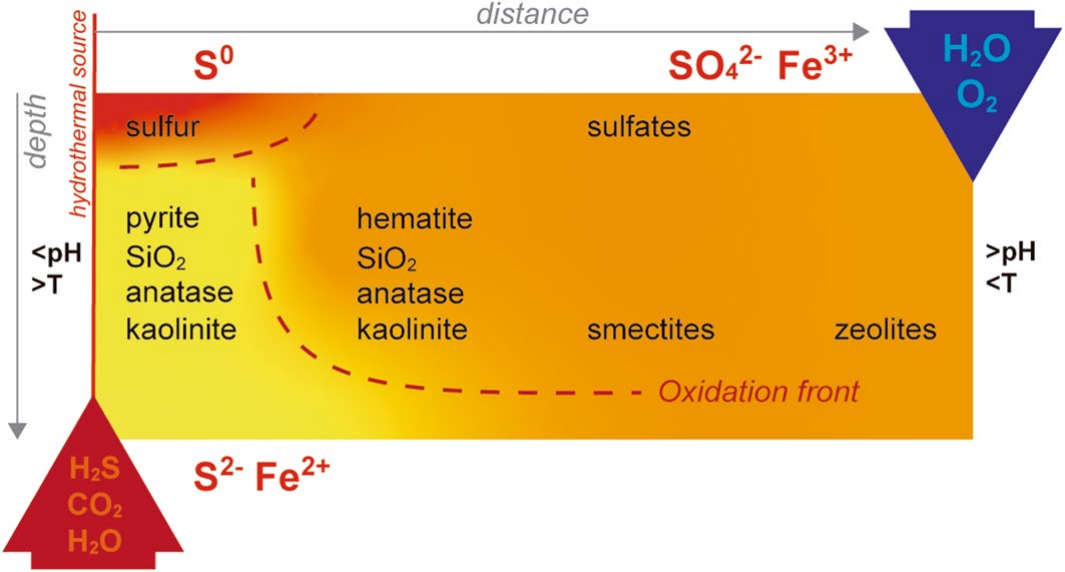
A simplified conceptual model of hydrothermal transect, showing the mineral distribution caused by hydrothermal alteration of original basaltic rocks at surface of the studied Iceland sites. Surface mineralogy is constrained by acid leaching and their exposure to oxidizing conditions, which depends on the physicochemical properties of hydrothermal (red arrow) and meteoric (blue arrow) fluids. Oxidation front (red dashed line) indicates the oxidation of sulfides and elemental sulfur into sulfates^20^.

The mineral sequence in these Icelandic substrates (Table 3) fits well with the mineralogical and geochemical trends produced in halos of geothermal surface alteration around mud pots and fumaroles^20^. Hydrothermal acid-sulfate alteration is a result of the interaction between aqueous H + and primary minerals (Fig. 6). The localized volcanic-derived high-temperature fluids enriched in H_2_S and CO_2_ produce extensive pyrite and native sulfur deposits (as found in samples MAT-78, MP-74, MP-87, IF-66, and IF-74). When they show low water–rock ratios conditions, they are considered gas fumaroles, while those with high water–rock ratios result in hot springs and mud pots. In the fumaroles, secondary minerals are generated from the volcanic vapors or during alteration of primary substrate, while in the mud pots they are controlled by the oxidation of iron sulfide deposits. The leaching dominates closest to the discharge source, resulting in significant cation mobilization, depletion of most major elements, and enrichment in SiO_2_ (e.g., amorphous phase or quartz) and TiO_2_ (e.g., anatase) (Fig. 6). Secondary iron and sulfur mineral assemblages are produced by their position along the oxidation front. In this way, sulfate-bearing solutions form sulfates such as natroalunite (as in AF-25) or iron-rich sulfates that breaks down to form hematite (as in IF-49). Smectite clays and zeolites are found in the distal margins of surface activity (as in MAT-54). This was supported by Raman spectra with peaks denoting alteration of minerals (e.g., native sulfur or hematite) primarily related to hydrothermalism (see Text S9).

Raman spectroscopy provided additional details about the formation conditions and potential biogenic origin of some mineral phases, besides detecting photosynthesis-related biomolecules (i.e., carotenoids) in MAT-54 (Text S9). For instance, in the identification of hematite in IF-49, the structural disorder suggested by the broad band around 800 cm^−1^ (Fig. 5) was interpreted to be potentially caused by both hydrothermalism or microbial intervention^42^ (see Text S9). In addition, the detection of certain allotropes of native sulfur combined with results from the other techniques used here, allowed constraining bioformation conditions^43,44^ in some samples (see Text S9). The coexistence of different allotropes of native sulfur was deduced from the position of its characteristic Raman peaks in the samples (Fig. 5;^45^). The α-sulfur allotrope mainly characterized by the Raman shifts around 220 and 473 cm^−1^—caused by the vibration of the internal modes of the S_8_ ring^46^—was observed to be dominant in lower-temperature samples such as IF-20 or IF-49 (Fig. 5). In contrast, Raman blueshifts of about 10 cm^−1^ related to the γ-sulfur allotrope were observed in higher-temperature samples such as MAT-78 and MP-87, from which a potential biomineralization source cannot be discarded^44^. In MAT-78, the substantial presence of SRB biomarkers (Fig. 2d) was consistent with a relative production of sulfur upon sulfate reduction that supports the idea that the sulfur cycle is essential in this system and is likely the main source of primary productivity in this biofilm.

Altogether, temperature played a role in conditioning the mineralogy that supports specific microbial metabolisms indirectly through the surface geothermal activity (i.e., in general, temperatures ≤ 70 °C revealed more alteration signs of the original basaltic petrology, and greater abundance of phototrophs and SRB biomarkers). Moreover, the generally sulfur-rich mineralogy of the substrates (mostly those at high temperature) and the abundance of biomarkers associated with microorganisms utilizing sulfur for their growth (e.g., SRB or green sulfur bacteria) revealed the importance of the sulfur cycle in the primary productivity of the Icelandic hydrothermal regimes.

## Conclusions

This work investigates mineralogical, molecular, and isotopic biosignatures on hydrothermal substrates from four regimes (hot spring biofilms, mud pots, active steaming fumaroles, and inactive fumaroles) to advance knowledge for the future search of molecular remnants on analogous Martian substrates. Whereas the study of the microbial ecology of hot spring biofilms has been previously addressed on Iceland, this is the first time that other hydrothermal substrates are investigated for their biomarkers fingerprints.

A direct comparison between Iceland and Martian hydrothermal systems is unachievable. Even when similar processes may have occurred, there are essential differences in water abundance, fluid chemistry, and atmospheric composition. This means that, for example, on Mars, soluble phases should be better preserved due to the lack of meteoric fluids (precipitation or other forms of surface water) whereas less preserved due to the intense irradiation on the Martian surface, and the dominant silica phase would be opal-A due to colder and drier conditions^47^. These opal-A rich deposits were found in Gusev crater by the Spirit Rover, whose wheel also exposed abundant sulfate minerals below the surface dust (^48^ and references therein), evidencing that these assemblages could be more widespread than previously thought. These deposits have even been associated with a fumarolic environment near the flanks of a volcano^10^, comparable to the Icelandic analog sites studied here, where amorphous silica was also detected.

Hydrothermal Iceland environments provide an interesting scenario to investigate habitability and biomarkers preservation for different reasons. First, the low complexity of the hydrothermal ecosystem on Iceland^24^ facilitates the interpretation of biosignatures associable with a few groups of microorganisms. Hot springs have even been singled out as one of the most favorable sites for the emergence of protocells^49^. Second, Icelandic landscapes combine a duality of glacial and volcanic features that offer a unique scenario to study habitability and adaptability to harsh environmental conditions. Third, all these features and the silica- and sulfur-rich mineralogy of the hydrothermal substrates convert Iceland on an accessible system to study biosignatures for its connections to Mars.

Molecular and isotopic distributions of lipids revealed compositional differences between the samples more or less influenced by mineralogical and geochemical factors (water supply and turnover, temperature, and pH). Overall, the results denoted the importance of the sulfur cycle in the hydrothermal substrates, representing an essential contribution to the primary production in these regimes. Besides, Raman spectral differences between samples suggested the possible coexistence of abiotic and biomediated sources of minerals (i.e., sulfur or hematite) in the Icelandic hydrothermal systems. Accounting for biomineralization is a major interest in the search of life on extraterrestrial substrates, where the scarcity of organics certainly hampers the detection of molecular (organic) biomarkers.

We seek mineralogical signatures associated with hydrothermal systems to identify potentially habitable locations on Mars. The findings of this study go further and shed light on the search of biosignatures on these geothermal systems with interest for its analogy to Mars (i.e., high sulfur content, hydrothermalism, or silica), providing insights about the best mineralogical assemblages for hosting biomarkers in sulfur- or silica-rich substrates. Our detection of lipids biomarkers, pigments, and potentially biomediated minerals by instruments (GC–MS and Raman spectroscopy) similar to those carried onboard the Perseverance (Raman spectroscopy Supercam) and Rosalind Franklin (Mars Organic Molecule Analyser and Raman Laser Spectrometer) rovers contributes to constrain the mineral context where biosignatures could be found in analogous scenarios on Mars (e.g., Gusev or Jezero craters). Understanding the relationship between biosignatures and inorganic variables such as mineralogy, temperature, redox state, or pH is vital for interpreting future Raman or GC–MS signals in the forthcoming Mars2020 (NASA) and ExoMars (ESA) missions to Mars, where the presence of organics is expected to be very low.

## Methods

### Study area

This study was conducted in three geothermal regions of Iceland distributed along the Mid-Atlantic Ridge; Krýsuvík, Hveragerdi (a.k.a. Hengill), and Námafjall (Fig. 1). On the north of Iceland, Námafjall is a high-temperature geothermal field (T above 200 °C at 1000 m depth) with a landscape defined by the homonymous hill and the abundant fumaroles, mud pots and hot springs (Fig. 1b) spread all over the base, slope and summit^21^, with abundant sulfur deposits. The hills substrate is primarily composed of basaltic hyaloclastite. Still, fumaroles in Námafjall are in contact with iron-enriched basalts at its bases. On the Reykjanes Peninsula (SW Iceland), the volcanic system of Krýsuvík is a basaltic territory made up of post-glacial lava fields, ridges of pillow lavas, and hyaloclastites^20^. The high-temperature geothermal area of Krýsuvík extends over 40–60 km^2^ and consists mainly of acid surface alteration and hot ground, steam vents, and steam-heated hot springs and mud pots (Fig. 1c). The surface geothermal activity in Krýsuvík affects its mineralogy strongly, with alteration mineral sequences varying from amorphous silica, anatase, pyrite, and native sulphur in areas of high activity (active steam vents and mud pots), to kaolinite and iron oxyhydroxides and oxides in areas of medium geothermal activity (hot grounds), and to montmorillonite on the margins of the surface geothermal activity^20^. Some 50 km northeastward, the Hveragerdi geothermal region (Fig. 1d) is in direct connection with the active Hengill volcano by fissures swarms^50^. Rocks in this area are composed of subglacially formed hyaloclastite and interglacial basalt from lava flows, whereas montmorillonite is the dominant alteration clay mineral at shallow depths. In Hveragerdi, geothermal waters are dominated by low salinity (mostly meteoric water), high sulphide concentration, high temperature, and alkaline pH^22,23^, although hydrothermal fluid from acidic to neutral pH (from 2.5 to 6.6) have also been reported^51^. Microbial mats in the sulphidic hot springs at Hveragerdi have been described to contain low species diversity compared to other hydrothermal regions^24^.

### Samples

In April 2018, a field campaign funded by the Europlanet Project was conducted to collect 11 samples of different hydrothermal substrates (biofilms, mud pots, and fumaroles) from three geothermal scenarios on Iceland (Fig. 1). In Námafjall, samples of mud and hot ground were collected from a grey mud pot at 74 °C (MP-74) and four inactive (i.e. non-steaming and with water contents ≤ 0.04%) fumaroles (IF) at temperatures ranging from 20° to 74 °C (Table 1), displaying different color (ochre, red, grey and pale yellow; respectively) (Fig. 1b). In Krýsuvík, a second mud sample was taken from another grey mud pot at 87 °C (MP-87) and two samples of hot ground from two active (i.e., steaming and with water contents ≥ 50%) fumaroles (AF) at 25° and 90 °C (Fig. 1c). In Hveragerdi, three biofilms or microbial mats (MAT) of different aspect (dark green, orange and light green, and light grey) were sampled at sites recording *in-situ* water temperatures of 54 °C (MAT-54), 70 °C (MAT-70), and 78 °C (MAT-78), respectively (Fig. 1d). The pH of all samples was measured in situ, and its values ranged from 1 to 2 in the MP and AF samples, from 1 to 4 in the IFs, and were 6 in the three MAT samples (Table 1). All samples were grabbed with solvent clean (methanol or MeOH and dichloromethane or DCM) stainless-steel spatula. They were all stored in solvent-cleaned (DCM and MeOH) polypropylene containers at − 20 °C and subsequently lyophilized. Before biogeochemical analysis, all samples were grounded in a pestle.

### X-ray diffraction (XRD), Fourier transform infrared spectroscopy (FTIR), and Raman spectroscopy

The mineralogical composition of the samples was determined by the combination of XRD and Raman spectroscopy. XRD was conducted using a Bruker X-Ray diffractometer (AXS D8-Focus), scanning samples in the 2 ϴ-diffraction angle from 5° to 70°, with a scanning step size of 0.01°, at 40 kV and 40 mA with a Cu X-ray source (Cu Kα1,2, λ = 1.54056 Å). Near Infrared (NIR; from 10,000 to 4,000 cm^−1^) spectra were collected at 4 cm^−1^ resolution with a Nicolet iS50FTIR spectrometer using the diffuse reflection (DRIFTS) attachment, a DTGS-KBr detector, and a XT-KBr beamsplitter. Raman spectroscopy was performed with a Horiba JobinYvon Hri550 connected to a Charge Coupled Device (CCD) with 1024 × 256 pixels cooled to 203 K for thermal-noise reduction. Before work, the Raman spectrometer was prepared with four diffraction gratings of 600, 1200, 1800 and 2400 grooves/mm that provide a wide range of intensity signal/resolution ratio. Sample excitation was done by an intensity-modulated (0–200 mW) non-polarized Nd:YAG solid state laser with a wavelength of 532 nm. The spectrometer was connected through fiber optics to a B&W Tek microscope with a 20X objective (Microbeam S. A.), which gives a spot size of 105 µm. When using a 1200 grooves/mm diffraction grating and 198 µm aperture of slit entrance, the resolution is better than 5 cm^−1^.

### Bulk organic geochemistry

Stable isotopic composition of total nitrogen (δ^15^N) and organic carbon (δ^13^C) was measured by Isotope-Ratio Mass Spectrometry (IRMS), using a MAT 253 (Thermo Fisher Scientific) and applying the USGS methods^52^, as described elsewhere^53^. Briefly, the homogenized, grounded samples were decarbonated with HCl and then dried (50 °C) after adjustment to neutral pH until constant weight. An analytical precision of 0.1‰ was determined by using three certified standards for carbon/nitrogen (USGS41, IAEA-600 and USGS40). The content of total nitrogen (TN) and organic carbon (TOC) was measured with an elemental analyzer (Flash HT, Thermo Fisher Scientific) during the stable isotope measurements.

### Molecular and isotopic lipid analysis

The lyophilized samples were extracted with a mixture of DCM:MeOH (3:1, v/v) to obtain a total lipid extract (TLE) by (i) ultrasonic bath (3x; further details in^54^) the MAT samples (1–3 g dw), or (ii) Soxhlet extraction (24 h; details in^55^) the rest of samples (~ 30 g dw). In all cases, internal standards (tetracosane-D50, myristic acid-D27, 2-hexadecanol) were added prior to extraction. The clean, concentrated and desulphurized TLE^54^ was hydrolyzed overnight with KOH (6% MeOH) at room temperature^56^. *n*-Hexane was added to the hydrolyzed TLE to obtain the neutral fraction through liquid–liquid extraction. The remaining lipidic extract was then acidified with HCl (37%) to remove K^+^ from the solution by precipitation of KCl, then recovering the liberated carboxylic groups by liquid–liquid extraction with *n*-hexane (acidic fraction). Further separation of the neutral fraction into non-polar (hydrocarbons) and polar (alkanols and sterols) was done according to a method extensively described elsewhere^55^. Aliquots of the acidic fraction were heated with 1 ml of 10% BF_3_ in MeOH at 80 °C for 30 min to methylate the fatty acids. Milli-Q water was then added and the products extracted with DCM (3x). Aliquots of the polar fraction were heated with 100 µl of N,O-bis(trimethylsilyl)fluoroacetamide (BSTFA) at 80 °C for 60 min to silylate the alcohols. Compounds in the apolar fraction (alkanes, alkenes and methyl alkanes) did not need of derivatization to be analyzed. All fractions were analyzed using gas chromatography-mass spectrometry (GC–MS) and isotope-ratio mass spectrometry system (IRMS) to determine the distribution and stable carbon isotopic composition of lipids (see^41^ for instrumental details). The identification of compounds was based on the comparison of mass spectra and/or reference materials on mass-to-charge ratios of 57 (*n*-alkanes and isoprenoids), *m/z* = 74 (*n*-fatty acids as fatty acids methyl esters or FAMEs), and *m/z* = 75 (*n*-alkanols and sterols). For quantification, we used external calibration curves of *n*-alkanes (C10 to C40), *n*-FAME (C8 to C24), *n*-alkanols (C10, C14, C18, and C20), and branched isoprenoids (pristane, phytane, squalane, and squalene), all from Sigma Aldrich. Recovery of the internal standards was measured to average 70 ± 21%.

### Statistical analysis

A principal component analysis (PCA) was performed with the software CANOCO5 v.5.12 (Microcomputer Power, Ithaca, NY) to understand the compositional variability of the hydrothermal samples (Text S7). For that, the distribution of nine variables was tested; biomass content (i.e., TOC), stable carbon isotopic composition of biomass (δ^13^C), and individual lipid biomarkers of bacteria, cyanobacteria, PhSnSB, SRB, archaea, and thermophiles. A second analysis based on Redundancy Analysis (RDA) was performed to assess the influence of selected environmental variables (pH, water, and temperature) on the microbial composition of the samples. In both PCA and RDA, the variables were centered and standardized prior to the analysis.

## Supplementary information

Supplementary Information.
