# Supplementary material for: Fingerprinting molecular and isotopic biosignatures on different hydrothermal scenarios of Iceland, an acidic and sulfur-rich Mars analog
Source: Sci Rep. 2020 Dec 3;10:21196. doi: 10.1038/s41598-020-78240-2 (PMC7712778; doi:10.1038/s41598-020-78240-2)
Supplement: Supplementary file 1 — Supplementary Information. [file 41598_2020_78240_MOESM1_ESM.docx]

# **Fingerprinting molecular and isotopic biosignatures on different hydrothermal scenarios of Iceland, an acidic and sulfur-rich Mars analog**

Laura Sánchez-García (1)*, Daniel Carrizo (1), Antonio Molina (1), Victoria Muñoz-Iglesias (1), María Ángeles Lezcano (1), María Teresa Fernández-Sampedro (1), Victor Parro (1), and Olga Prieto-Ballesteros (1)

**Supplementary Material**

This section contains 9 supplementary texts and 10 figures**.**

**Text S1. Source assessment based on lipid biomarkers: use and limitations.**

The abundance and distribution of biomarkers represent a mixture of contributions from all organisms present in the environment, with certain molecular biomarkers being unique and diagnostic for specific microorganisms that can provide insight on overall community structure and biogeochemical processes occurring at a location [1]. In the Icelandic hydrothermal regimes, a number of lipids and their ratios were used as geochemical proxies to infer likely input sources, relative proportion of microbial groups, or extent of biological activity.

On the one hand, a number of lipid biomarkers considered diagnostic of more or less specific sources was used to estimate the contribution of certain microbial sources (*i.e.* bacteria, cyanobacteria, sulfate-reducing bacteria or SRB, photosynthetic sulfur and non-sulfur bacteria or PhSnSB, archaea and thermophiles). For a general estimate of **bacterial** biomass, we employed the amount of low molecular-weight (LMW) fatty acids [2] as the sum of saturated straight-chain (*normal*) fatty acids (*n*-fatty acids) from 16:0 to 18:0 [3], whereas for that of **archaea** we used squalene, an isoprenoid considered a biomarker for archaea, either methanogenic, halophilic or thermoacidophilic [4, 5]. To assess the proportion of **cyanobacteria** we considered the aggregated amount of *n*-heptadecane, isomeric *n*-heptadecenes, monomethyl alkanes (MMA) of C_17_, C_18_ and C_19_, [6, 7, 8, 9], diploptene [10], and the 16:1ω7, 18:2ω6, and 18:3ω6 fatty acids [9, 11, 12]. The participation of **SRB** was inferred from the sum of lipid biomarkers such as phytane [13], the branched fatty acids *i/a*-15:0, *i/a*-17:0, and *i/a*-15:1 [14], or the monounsaturated fatty acids 16:1ω5, 17:1, and 18:1ω5 [14, 15, 16]. Phytane is an isoprenoid mostly derived from the chlorophyll side chain phytol [17] that typically forms from its transformation in anoxic conditions [18] such as upon sulfate reduction. Finally, the proportion of **PhSnSB** was deduced from the sum of the *n*-alkanols C_16_, C_17_, and C_18_ [19], whereas that of **thermophiles** from the abundance of dicarboxylic acids [20]. Despite being just an estimate (see limitations below), these biomarkers provide a general comparative view of the relative abundance of certain major microbial groups in the different hydrothermal regimes (Fig. 2).

On the other hand, different lipid ratios are used as proxies of the relative abundance of biomass from certain microbial groups. For instance, the proportion of *n*-heptadecane relative to *n*-hexadecane and *n*-octadecane (*i.e.*, *n*-C_17_/[*n*-C_16_+*n*-C_18_]) was employed to estimate the fingerprint of cyanobacteria over other bacteria [21]. The ratio of branched over *normal* heptadecane (br-C_17_/*n*-C_17_) is a proxy of the relative abundance of heterotrophs [22]. Finally, the ratio of *n*-fatty acids over *n*-alkanes may be used as a sign of freshness or extent of biological activity, since oxygen-functional groups tend to undergo dehydration and decarboxylation during diagenesis [23], thus resulting into saturated hydrocarbons (*i.e.*, *n*-alkanes) over time.

Note that the aim of this study was not to exhaustively account for all biological sources contributing to the organic matter in the Icelandic samples, but rather comparing their relative abundance in certain biosources by detecting a number of widely used lipid biomarkers. Thus, not all existing sources contributing to the sample biomass were considered, such as eukaryotic. In spite of the detection in the samples of a few lipid compounds associated to eukaryotes (*e.g*., phytosterols or odd and high-molecular weight alkanes, from plants; or cholesterol and derivates, from animals), their contribution to the hydrothermal substrates was not accounted for here, given the astrobiological scope of the study (*i.e*., in case of existing life remnants on Mars, it is more likely that it was of simple nature, that is prokaryotic). Furthermore, the potential eukaryotic origin of other lipid compounds widely present in all biological sources, such hexadecenoic (*i.e*., *n*-16:0) and octadecanoic (*i.e*., *n*-18:0) fatty acids, was discarded here because of the virtual absence of fatty acids of high molecular weight (i.e., >C_20_). The exclusive presence of low-molecular weight acids is commonly related to bacteria [2].

The source assessment approach based on lipid biomarkers is commonly employed in geochemical studies, and has been largely employed in environmental samples of different nature [*e.g*. 21, 24, 25], based on the detection of a number of generally accepted source biomarkers [17]. Its potential limitations may come mostly from the limited taxonomic specificity that lipids provide compared to other biomarkers such as DNA, although, as a counterpart, lipids offer a relatively higher preservation potential over time. As structural components of cell membranes, lipids are rather ubiquitous in all organisms, thus producing molecular fragments upon extraction that, in some cases, are widely present in most biological sources. That is the case of straight chain aliphatic families (*i.e*. *normal*) such as *n*-alkanes, *n*-fatty acids, or *n*-alkanols, which are widely detected in samples of different sources. Still, the molecular distribution pattern of the series and the relative abundance of certain moieties over others in a given series, provide information about the dominance of one or another source. Thus, what it is relevant when interpreting biosignatures from the *normal* lipidic series is not as much the individual detection of a particular compound, but instead the modalities observed in the molecular distribution of the series and the maximum peak/s governing them. For instance, when a compound like heptadecane is observed to clearly stick out the *n*-alkanes distribution, it is generally interpreted as an input of cyanobacterial biomass. Besides, the inference of a certain biosource in a sample is generally based on the simultaneous detection of various biomarker compounds, (*e.g*., middle-chain MMA, plus heptadecane, plus diploptene, plus the 16:1ω7, 18:2ω6, and 18:3ω6 fatty acids, for cyanobacteria), not only one. This way we reduce the likelihood of accounting for a biological source not really present in a sample.

Still, there are limitations to be aware of when interpreting biosignals from lipid analysis, which may be considered to potentially come from two directions. On the one hand, overestimation of certain sources (*e.g*., bacteria, SRB, or PhSnSB here) may come from the limited specificity of the biomarkers considered (*i.e*., *n*-fatty acids from 16:0 to 18:0, for bacteria; *i/a*-pairs of 15:0, 17:0, and 15:1 fatty acids, for SRB; or C_16_, C_17_, and C_18_ *n*-alkanols, for PhSnSB) that are not exclusive (but characteristic or dominant) of the presumed sources. On the other hand, underestimation of any biosource may come from involuntary disregarding of biomarkers that, despite present in the sample, are not detected because of insufficient analytical precision (detection limit) or technical expertise. However, as mentioned above, the basis of the lipid-biomarkers approach is not to comprehensively and quantitatively account for all the biological sources contributing to the sample biomass, but rather to identify major biosources and compare relative abundances between samples. While the real and absolute contribution of each biological source to each sample is not critical, an estimate of the relative abundance of each source is important to assess which biological groups dominate the community structure in each sample/scenario. Therefore, considering the semiquantitative interpretation of the data and assuming that the error derived from the mentioned limitations likely affects all samples in a similar way, we consider that the present source assessment based on the use of the mentioned lipid biomarkers is adequate for the main goal of this study (*i.e*., identifying characteristic lipid biosignatures in different hydrothermal substrates in relation to their local mineralogy and physicochemistry).

**Text S2. Source and carbon metabolism from stable carbon isotopic composition**

Organisms capable of autotrophic metabolism assimilate inorganic carbon into organic carbon to build their cell material. Microbial pathways for autotrophic carbon fixation are mainly four, two involving great ^13^C fractionation such as the Calvin cycle (Δ≈10-22 ‰; [26]) and the reductive acetyl-CoA pathway (Δ≈20-36 ‰; [26, 27]), and two discriminating less against ^13^C, such as the 3-hydroxypropionate (3HP) bicycle (Δ≈2-13‰ [28]) and the reductive tricarboxylic acid (rTCA) pathway (Δ≈4-13‰ [26, 27]). Biomass produced by microorganisms using the mentioned carbon acquisition mechanisms typically shows bulk δ^13^C ratios ranging from -19‰ to -30‰ (Calvin cycle), from -28‰ to -44‰ (reductive acetyl-CoA pathway), from -12‰ to -21‰ (rTCA), or from -4‰ to -15‰ (3-HP) [26, 27, 28].

The four carbon fixation pathways distribute among major phylogenetic lineages, bacteria and archaea, in such a general way [29]: *Cyanobacteria* and β-*Proteobacteria* are groups fully using the Calvin cycle, as well as most α- and γ-*Proteobacteria*, and certain *Firmicutes* and *Chloroflexi*; *Euryarchaeota*, *Spirochaetes*, *Planctomycetes* and acetogen *Firmicutes* rather use the reductive acetyl-CoA pathway; *Chloroflexi* is the only phylum using the 3HP bicycle; *Nitrospirae*, *Aquificales*, *Chlorobiales* and ε-*Proteobacteria* fix inorganic carbon through the rTCA pathway, as well as some α-, γ-, and δ-*Proteobacteria*; whereas *Crenarchaeota* use minority variants of the 3HP bicycle (*i.e.*, the 3-hydroxypropionate/4-hydroxybutyrate or the 3-HP/4-HB, or dicarboxylate/4-hydroxybutyrate or DC/4-HB).

In the studied Icelandic hydrothermal regimes, stable carbon isotope analysis was conducted both on the total biomass (*i.e.* bulk δ^13^C) and on individual lipid compounds (*i.e.*, compound specific δ^13^C). For the latter, individual lipids must be present in sufficient concentration as to be detected by the IRMS instrument. In cases of low biomass content (*i.e.*, total organic carbon or TOC), individual lipids may be not sufficiently abundant for measuring their specific carbon isotopic composition, such as in the inactive fumaroles (IF) here. Consequently, in those samples, only the bulk δ^13^C was then determined.

**Text S3. Molecular lipid patterns on the three Icelandic hot spring biofilms**

In the hot springs, the molecular distribution of lipids showed different patterns in the three biofilms. In the **dark green MAT-54**, the apolar fraction, majorly composted of *n*-alkanes from C_14_ to C_30_, was dominated by low molecular-weight (LMW) moieties (Fig. S1 a), mostly *n*-heptadecane (C_17:0_). Other apolar compounds such as isomeric *n*-heptadecenes (C_17:1_), or middle-chain MMAs of C_15_ and C_17_, or diploptene were also detected at low concentrations. The acidic fraction was dominated by the even LMW *n-*fatty acids 16:0 and 18:0, together with their mono- (16:1 and 18:1) and polyunsaturated (16:4, 18:2, 18:3, 18:4) homologues (Fig. S1 d). Similarly, the polar fraction was also dominated by the C_16_ and C_18_ *n*-alkanols, as well as a comparable amount of neophytadiene and, to less extent, phytol (Fig. S1 g).

In the light **green-orange MAT-70**, *n*-C_17_ was the most abundant compound in the apolar fraction, which also contained a relatively large proportion of monounsaturated fatty acids (16:1, 17:1, 18:1, and iso-15:1) and middle-chain MMA of C_17_, C_18_, and C_19_. Diploptene was similarly abundant as in MAT-54, but squalane was about 4-fold more abundant in MAT-70 than there (Fig. S1 b). The acidic profile was also slightly different than that in MAT-54. Similar to that, MAT-70 had a maximum peak at 16:0, but it was followed by 14:0 and a relatively greater variety of mono- (from 16:1 to 20:1) and polyunsaturated (16:4, 16:3, 16:2; 18:4, 18:3, 18:2; 20:5, 20:4) acids (Fig. S1 e). In addition, the polar fraction in MAT-70 showed a relatively larger proportion of high molecular-weight (HMW) *n*-alkanols (C_24_, C_26_, and C_28_), phytol, neophytadiene and vegetal sterols (Fig. S1 h). Particularly high was the concentration of phytol (46 µg·g^-1^ dw), which was 3-4 orders of magnitude greater than in the other two mats.

In the **grey MAT-78,** new lipid features were detected together with some other shared with MAT-54 and MAT-70. The apolar fraction showed a bimodal profile, with the distributions of LMW and HMW *n*-alkanes dominated by C_17:0_ and C_27:0_, respectively. A low presence of squalene was observed, in contrast to that of diploptene (Fig. S1 c). The acidic fraction was the least abundant of the three biofilms, with a maximum peak at 16:0, lower and similar concentration of 18:0 and monounsaturated compounds (16:1, 18:1, and 20:1), and minor presence of 18:4, *iso*-15:0, *iso*-17:0, and cyclopropane heptanoic acid (Cy17:0) (Fig. S1 f). The polar fraction similar distributions of LMW and HMW *n*-alkanols dominated by C_16_ and C_26_, respectively, as well as neophytadiene (Fig. S1 i).

In MAT-54 and MAT-70, the relative abundance of lipids such as *n*-heptadecane and isomeric *n*-heptadecenes, middle-chain MMAs of C_17_ [6, 7, 8, 9], mono- (16:1ω7 and 18:1ω9) and polyunsaturated (18:2ω6 or 18:3ω6) fatty acids [9, 11, 12], or diploptene [10] led us to consider that cyanobacteria contributed significantly to the microbial community in these samples. In fact, these compounds were already reported in photosynthetic microbial mats from Hveragerdi hot springs collected from waters between 44º and 66ºC [30], where the thermophilic *Mastigocladus* was observed by microscopy to be dominant. *Mastigocladus* is a cyanobacterial genus inhabiting the majority of Icelandic thermal streams, together with other cyanobacteria from the *Oscillatoria* or *Phormidium* genera [31]. The highest temperature tolerance of *Mastigocladus* is thought to be ~64ºC, but the limit may be higher (<74ºC) for other cyanobacteria [32]. According to this, the observed temperature of 70ºC in MAT-70 was above the reported tolerance of *Mastigocladus*, however, the water temperature over these mats may be lower at certain periods of the year, allowing colonization by methyl alkane-producing cyanobacteria [30]. Another possibility is the cohabitation of *Mastigocladus* with more temperature-tolerant microorganisms such as *Chloroflexus*, which buffers the excess of temperature and sulfide adverse for the cyanobacterim by surrounding the *Mastigocladus* mat. Thus, the *Mastigocladus* mat is commonly surrounded by or underneath that of *Chloroflexus*, which removes sulfide by anoxygenic photosynthesis (*i.e.* using sulfide as electron donor) and allows the otherwise inhibited growth of the cyanobacterium [33]. The *Mastigocladus*-*Chloroflexus* cohabitation has been described to occur in the Icelandic thermal streams, with dense bright orange mats of *Chloroflexus* (similar to our MAT-70) typically form upstream the green *Mastigocladus* mats (similar to our MAT-54), where water temperature exceeds its temperature limit [30, 34].

Compared to MAT-54 and MAT-70, the proportion of cyanobacteria and phototrophs seemed much lower in MAT-78. Indeed, the proportion of *n*-heptadecane over *n*-hexadecane and *n*-octadecane in MAT-78 (2.7) was considerably lower than that in MAT-54 and MAT-70 (75 and 12, respectively), reflecting the comparatively lower presence of cyanobacteria in the former sample [21]. Accordingly, the molecular distribution of lipids in this mat was considerably different from that in the other two mats (*i.e.* lower proportion of *n*-heptadecane, *n*-heptadecenes or middle-chain MM-C_17:0_; relatively higher abundance of monounsaturated over polyunsaturated acids; Fig. S1). In contrast, the relative abundance of monounsaturated (16:1ω5 and 18:1ω8 [35]) as well as *i/a-* fatty acids (15:0 and 17:0) with a predominance of *iso* over *anteiso* moieties [14] in MAT-78 are compatible with a relatively larger presence of sulfate-reducing bacteria or SRB (*e.g.* *Firmicutes* or δ-Proteobacteria) in this compared with the other two mats.

In addition to the microbial sources, the contribution of organic matter from algae and the surrounding vegetation to the hot spring biofilms was inferred from the presence of polyunsaturated fatty acids (16:3, 16:4; 18:4; 20:4, 20:5 [11, 36]), vegetal sterols [37], as well as HMW *n*-alkanes, *n*-fatty acids and *n*-alkanols [38] (Fig. S1). The vegetal input source to the phototrophic biomass was particularly high in MAT-70, where there was a great concentration of vegetal sterols (phytosterols) such as campesterol, stigmasterol and β-sitosterol as well as a dominance of phytol (Fig. S1h).

**Text S4. Bulk and compound-specific stable carbon isotopic composition of the Icelandic biofilms**

The stable carbon isotope (bulk and molecular) analysis of the three biofilms may help to constrain their microbial composition, since different pathways for incorporating inorganic carbon produce different isotopic signatures (Text S2). In the present study, the stable carbon isotope composition of the three Icelandic biofilms reflected contributions from mixed carbon fixation pathways, with different interpretations depending on the mat. In **MAT-54 and MAT-70**, the stable carbon isotopic composition of bulk biomass and lipids suggested contribution of mixed pathways for fixing inorganic carbon, compatible with a microbial community dominated by *Chloroflexus* and cyanobacteria. On the one hand, compound-specific δ^13^C ratios varied from values as enriched as -18‰ to values as depleted as -30‰ (Fig. S2). On the other, both bulk δ^13^C were similarly enriched in ^13^C (*i.e.* -18.2‰ and -17.9‰, respectively; Table 2). Cyanobacteria typically incorporate atmospheric CO_2_ using the Calvin cycle that produces biomass isotopic signatures from -18‰ to -30‰ [10, 26], whereas green non-sulfur bacteria such as *Chloroflexus* fixes inorganic carbon (HCO_3_^-^) through the 3HP pathway [28], thus producing organic carbon enriched in ^13^C by ~14‰ relative to the Calvin Cycle (*i.e.* δ^13^C from -4‰ to -15‰; 28, 39]). In the presence of cyanobacteria, the growth of *Chloroflexus* has also been described to be photoheterotrophical [40], but the stable carbon isotopic composition observed here led us to rule out that option. When *Chloroflexus* grows heterotrophically on cyanobacterial products [40] using light energy to assimilate them [41], it is cross-fed by ^13^C depleted organic compounds from cyanobacteria, thus producing biomass with values of δ^13^C similarly depleted as those of cyanobacteria (*i.e.*, Calvin cycle). However, in this case, both δ^13^C ratios (*i.e.*, bulk and lipids specific) in MAT-54 and MAT-70 showed values in between those typical of 3HP (*i.e.*, enriched) and Calvin (*i.e.*, depleted) signatures, thus supporting the autotrophic growth of *Chloroflexus*. In fact, values of the bulk δ^13^C in MAT-54 and MAT-70 (-18.2‰ and -17.9‰, respectively) were slightly ^13^C depleted than those measured on pure *Chloroflexus* mats (-14.9‰) growing autotrophically in similar hot springs from Yellowstone National Park [39].

**In MAT-78**, the bulk and compound-specific isotopic composition also denoted mixed mechanisms for carbon acquisition, although with different interpretations according to its different microbial composition relative to MAT-54 and MAT-70. Compared to them, the bulk ^13^C content in MAT-78 (δ^13^C of -20.8‰) was slightly more depleted (Table 2), and the compound-specific δ^13^C ratios varied more widely from -19.1‰ to values as low as -34.3‰ (Fig. S2). These results suggested important participation of carbon fixation pathways involving large fractionation of ^13^C (*i.e.*, Calvin cycle or reductive acetyl-CoA pathway). The high temperature of the water in MAT-78 ‒over the upper limit of chlorophyll a (*i.e.*, 74ºC [32])‒ and the different lipid profile observed here compared to the other two mats, led us to consider a minority presence (or total absence) of phototrophs in MAT-78 (see sample aspect in Fig. 1d). Therefore, although the absolute absence of phototrophs cannot be excluded entirely due to the variability of the water temperature during the year [30], the dominance of Calvin and/or 3HP pathways was generally discarded in detriment to other carbon fixing mechanisms. Input sources of microorganisms commonly using the reductive acetyl-CoA pathway such as *Firmicutes* or δ–*Proteobacteria* [29] was supported by the relative abundance of compounds diagnostic of gram-positive bacteria [42] and SRB [14, 43], such as *i/a-* pairs of 15:0 and 17:0 fatty acids (Fig. S1f). The use of the reductive acetyl-CoA would explain the most depleted δ^13^C values observed in this sample (Fig. S2). In addition, certain participation of rTCA fixers could also play a role in explaining the relatively enriched bulk δ^13^Crelative to typical reductive acetyl-CoA values. Among the microbial groups described in similarly high-temperature hot springs from Hveragerdi [44], members of *Aquificales* or *Nitrospira* are known to fix carbon through the rTCA [29].

**Text S5. Molecular lipid patterns in the Icelandic mud pots and fumaroles**

Molecular lipid profiles in the rest of geothermal substrates suggested microbial community structures different from those in the hot spring biofilms. Overall, the general dominance of the apolar fraction by the *n*-heptadecane (or the isomeric *n*-heptadecenes) was no longer observed in neither the MP, AF nor IF substrates (Fig. S3-S5). Similarly, typical cyanobacterial mono- (16:1ω7) and polyunsaturated (16:2ω7, 18:2ω6, and 18:3ω6) fatty acids, relatively abundant in the hot spring biofilms (mostly in MAT-54 and MAT-70), were observed to be rarely present in the rest of substrates. In contrast, other compounds gained importance in these substrates lipidic profiles.

In the **mud pots**, HMW *n*-alkanes with a general odd-over-even predominance contributed largely to the distribution of the apolar lipids, together with squalene in MP-74, or pristane and mostly phytane in MP-87 (Fig. S3 a-b). The acidic fraction showed a relative abundance of *i/a-*pairs of 15:0 and 17:0, and monounsaturated 16:1ω5 and 18:1ω8 fatty acids mostly in MP-74 (Fig. S3 c-d). These compounds are considered biomarkers of sulfate-reducing bacteria (SRB; see Text S1) consistent with the active degradation of phytol to phytane and neophytadiene (Fig. S3 e-f) by SRB [13].

In the **active fumaroles**, the mostly microbial C_16:0_ and C_18:0_ *n*-alkanes dominated the apolar fraction, together with the generally vegetal C_29:0_ mostly in AF-90 and the isoprenoids pristane and phytane mostly in AF-25 (Fig. S4 a-b). The AF acidic fractions were dominated by the 16:0 fatty acid, with less proportion of 18:0, and the typically SRB biomarkers 16:1ω5 and 18:1ω8, as well as *i/a-*pairs of 15:0 and 17:0 only in AF-25 (Fig. S4 c-d).

In the **inactive fumaroles**, a bimodal distribution of *n*-alkanes with maximum at C_16:0_ or C_18:0_ and C_25:0_, C_27:0_ or C_29:0_ represented the distribution of the apolar lipids, with additional relevance of phytane and MM-C_17:0_ (Fig. S5 a-d). The acidic fraction was largely represented by fatty acids such as 16:1ω5 18:1ω8, and *iso*-18:0, as well as dicarboxylic acids (dioics; Fig. S5 e-h). The dicarboxylic acids, although generally detected in all substrates, was observed to be relatively more abundant in the active (Fig. S4) and (mostly) inactive fumaroles (Fig. S5).

In order to compare the microbial composition of the different regimes and their samples, the relative proportion of input biosources based on a number of lipid biomarkers diagnostic of more or less specific sources (Text S1) was represented in Fig. 2. The presence of cyanobacterial biomarkers was negligible in the mud pots and fumaroles, consistent with the low pH in all these substrates, typically inhibiting the growth of cyanobacteria at values below 4 [45]. In contrast, other microorganisms were more relatively more abundant in these substrates, such as thermophiles in the IF´s, or bacteria, archaea, SRB and PhSnSB in the MPs and AFs (Fig. 2). Heterotrophic biosignatures (branched over *n-*heptadecane ratios > 1; see Text S1) were stronger in MP-87 and AF-90 than in the rest of samples (Table 2). This could be related to the presence SRB such as members of *Nitrospira*, *Crenarchaeota*, or *Thermodesulfobacterium*, as well as fermentative bacteria (*Thermotogales*) described by others on Hveragerdi [46].

**Text S6. Bulk and compound-specific stable carbon isotopic composition of the Icelandic mud pots and fumaroles**

The stable carbon isotope composition of the hydrothermal substrates other than biofilms also denoted compositional differences compatible with the participation of different carbon acquisition mechanisms.

In the **MPs**, the wide range of bulk (from -16.1‰ to -21.2‰; Table 2) and compound-specific (from -15.5‰ to -34.0‰; Fig. S6) δ^13^C values suggested the mixed contribution of carbon fixation pathways with large and small fractionations. The generally depleted compound-specific δ^13^C values pointed to dominance of carbon fixation mechanisms involving large ^13^C fractionation (*i.e.*, Calvin cycle and/or reductive acetyl-CoA pathway). At the range of temperatures operating in the MP regime (*i.e.*, ~70-90ºC), microorganisms using the Calvin cycle could be γ- or β-Proteobacteria, as well as cyanobacteria in the lower limit of the temperature range, whereas those fixing carbon through the reductive acetyl-CoA pathway may include *Euryarchaeota* (methanogenesis), *Archaeoglobales* (sulfate reduction) or δ-Proteobacteria (sulfate reduction) [29]. Considering the lipid biomarkers-based microbial composition (Fig. 2), the depleted δ^13^C signatures in MP-74 could be attributed to the use of the Calvin cycle (*i.e.*, higher abundance of cyanobacteria and PhSnSB), whereas those in MP-87 could be rather influenced by the reductive acetyl-CoA pathway given the relative abundance of archaeal biomarkers. In addition, the larger proportion of PhSnSB (including *Chlorobiaceae* and *Chloroflexi*) biomarkers in MP-74 may explain its relatively enriched bulk δ^13^C (-16.1‰) compared to that in MP-87 (-21.2‰). Both *Chlorobiaceae* and *Chloroflexi* employ carbon fixation mechanisms (*i.e.*, rTCA and 3HP, respectively) applying large discrimination against ^13^C, thus producing ^13^C-enriched biomass (*i.e.* δ^13^C from -12‰ to -21‰ [26]; or from -4‰ to -15‰ [39]; respectively). Other potentially users of the rTCA pathway typically inhabiting hydrothermal vents at temperatures between ~70º and 90ºC may be *Aquificales* [29]. Despite our lack of detection of *Aquificales*-specific lipid biomarkers, the presence of these thermophiles in the MP regime is not unreasonable given their previous report in other Icelandic substrates [44, 46] and the high temperature in both MP substrates, which may be connected to the detection of thermophiles biomarkers such as dicarboxylic acids (Fig. S3 c-d).

In the **AF** regime, the range of compound-specific δ^13^C values (from -20.0‰ to -33.2‰; Fig. S7) was similar to that in the MPs, whereas the bulk δ^13^C ratios were relatively more enriched (Table 2), mostly in AF-90 (-8.0‰). This suggests a similarly mixed participation of different carbon fixation routes, involving i) a dominant reductive acetyl-CoA pathway (consistent to the relative abundance of archaea and/or SRB) that causes large fractionation of ^13^C (Text S2) and thus more negative δ^13^C ratios (Fig. S7), and ii) a minority contribution from the 3HP bicycle (*Chloroflexi*) and/or rTCA (*Aquificales*, *Nitrospira*, or *Chlorobiaceae*), both producing less negative δ^13^C (Text S2).

In the **IF**s, the enriched values of bulk δ^13^C (from -10.0‰ to -21.1‰; Table 2) ‒the only isotopic analysis accomplished in these samples‒ led us to hypothesize a meaningful participation of the highly fractionating pathway rTCA, compatible with the relative abundance of thermophile biomarkers the IFs compared to the rest of substrates (Fig. 2f) and the presence of PhSnSB (Fig. 2c).

**Text S7. Organic compositional differences between substrates**

The microbial community structure based on the molecular and isotopic composition of the sample varied in the Icelandic hydrothermal scenarios within substrates. To statistically assess the compositional variability of the samples, a Principal Component Analysis (PCA) was conducted using nine organic measures as variables (Fig. S8). The variability of the samples´ composition was explained by two principal components together accounting for ~72% of the total variance. In the PCA plot, the samples were distributed in three separated groups (MAT-70; MAT-54 and MAT-78; and rest of samples). The samples showing the lowest biomass (*i.e*., mud pots and fumaroles) clustered together and showed a negative correlation with TOC, whereas the three hot spring biofilms were plotted separately in the PCA diagram based on their different microbial composition and abundance. MAT-70 showed relatively higher contribution from cyanobacteria, PhSnSB, or archaea, and MAT-54 and MAT-78 were rather enriched in SRB (Fig. 2). The rest of samples (mud pots and fumaroles) appeared to be rather related to the presence of thermophiles and the stable carbon isotopic composition (δ^13^C). In particular, the latter variable was close to IF-66 and AF-90, suggesting similarity of both samples in terms of δ^13^C. The less negative δ^13^C observed in these samples (*i.e*., -8/-10‰) compared with the rest of samples (*i.e*., ≤ -16‰) suggested greater incorporation of inorganic carbon through the 3HP and/or rTCA pathways in the two samples relative to the others.

**Text S8. Environmental variables affecting the biological fingerprints in the Icelandic substrates**

The influence of environmental variables on the organic compositional variability of the Icelandic hydrothermal samples was assessed with a Redundancy Analysis (RDA), using water, pH, and temperature as predictors (Fig. 4). Global permutation test indicated a linear relationship between the environmental variables and the microbial composition of the samples (p=0.012). Most of the composition variability was explained by the first axis (44.34%), and the two axis together accounted for the 50.42%. pH and water showed a positive correlation with TOC and most biomarkers (*i.e.*, archaea, bacteria, PhSnSB, cyanobacteria, and SRB), while temperature barely explained compositional differences attending to a vector angle relative to those of ~90º (Fig. 4). In contrast, temperature and water showed a negative correlation with thermophiles, and thus most active and inactive fumarole samples were located separately from the rest of the samples in the ordination plot.

As for **water**, biomass was highest in the hot spring biofilms containing the highest proportions of water (93-98 %), followed by the MPs (52-82%) and AFs (57-71 %), and ultimately by the virtually dry IFs (0.02-0.04 %) (Fig. 3a). Similarly, higher biological activity measured as the ratio of *n*-fatty acids over *n*-alkanes (see Text S1) was generally found in samples with relatively greater water content (Fig. 3b). According to this, the most hydrated (*i.e*., the three biofilms or mats) clustered together at the right half of the RDA diagram and closely related with the water variable (Fig. 4). However, the most relevant aspect is likely not as much the water abundance itself, but its exchange rate in the sample setting, where continuously renewed running water produced higher biomass in the hot spring (*i.e*. biofilms) than less renewed or stagnant water in the mud pots, scanty steaming vapor in the active fumaroles, or virtually absent water in the inactive fumaroles.

The **pH** variable also showed an influence on the microbial composition of the Icelandic samples, both at quantitative and qualitative level. On the one hand, the higher the pH (max. of 6 in this study) the higher the biomass content (*i.e*. TOC) in the samples (Table 1 and Fig. 3a). On the other hand, samples with the highest pH (*i.e*., 6 in the three biofilms) contained the greatest concentration of biomarkers related to bacteria, cyanobacteria, and SRB in general, as well as to PhSnSB and archaea in the case of MAT-70 (Fig. 2). Accordingly, the three biofilms clustered together in the RDA plot close to the pH variable (Fig. 4). The growth of some microorganisms may be directly affected by the environmental pH, such as cyanobacteria, which growth is generally inhibited at values lower than 4 [32]. Overall, in the range of values considered in the present study (*i.e.,* from 1 to 6), the greatest biomass is produced in settings with circumneutral pH and highest supply and turnover of water.

In contrast to water and pH, **temperature** showed lower influence on most of the microbial biomarkers and on the total biomass according to the RDA (Fig. 4). Only thermophiles biomarkers resulted negatively correlated with temperature, with strongest signal of thermophiles being detected at IF-20 (Fig. 2f). This and the lack of correlation between most biomarkers with temperature in the IF regime was interpreted in relation to the thermal instability of hydrothermal systems with time [47]. Thermal activity in such systems is known to be episodic, with continuous transitions between inactivity and actively emitting steam [48]. Here, the inactivity of the fumarole regime deduced from the absence of steaming during the samples collection was confirmed by the low water content measured in the four IF samples (Table 1). As for the temperature, the values measured in the IF substrates at the time of collection were considered to correspond to punctual values along the fumarole lifetime (with likely different values in the recent past), rather than steady values over time. This typically episodic nature of temperature converts any study of hydrothermal systems in a snapshot in time, where the punctual detection of biomarkers might reflect combined information of both present and past microbial inhabitants [49, 50]. Thus, the highest detection of thermophile biomarkers in the inactive fumarole at ~20ºC (Fig. 2f) was thought to be likely recording a larger presence of thermophiles in the past, when a much higher temperature than that measured during the study sampling surely took place.

**Text S9. Mineral composition of Icelandic sulfur-rich hydrothermal substrates**

X-Ray Diffraction, Raman Spectroscopy, and Fourier Transform Infrared spectrometry (FTIR) were applied to characterize the inorganic fraction of the Icelandic samples. Results from all the three analyses are complementary; whereas XRD better detected crystalline minerals, spectra in the near and mid infrared confirmed the presence of amorphous phases, and Raman spectroscopy verified the mineralogy at microareas.

**XRD and Raman** results revealed that the sampled Icelandic hydrothermal substrates were dominantly composed of anatase (TiO_2_) and sulfur minerals (Table 3 and Fig. 5), together with amorphous silica (SiO_2_), which was corroborated by **FTIR** measurements in the near infrared range (1-2.5 microns; Fig. S9). In the hot spring biofilms, crystallized forms of inorganic matter were only detected in MAT-54 and MAT-78 (Table 3), where mineralogy was dominated by heulandite and montmorillonite in the former, and by pyrite (FeS_2_) and elemental sulfur (S_8_) in the latter. In the rest of hydrothermal substrates, the variety of minerals detected included elemental sulfur, pyrite, and anatase, in barely all samples.

Other secondary minerals found in certain samples were kaolinite in MP-74, MP-87, and AF-25; hematite in IF-49; natroalunite in AF-25: and quartz in IF-20 (Table 3). The presence of hematite in IF-49 was confirmed by Raman analysis (Fig. 5). Overall, minerals rich in sulfur (*i.e.*, pyrite or elemental sulfur) dominated at sites with high temperatures (≥66°C), whereas other phases such as hematite or clay minerals (kaolinite, natroalunite, montmorillonite, or heulandite) were progressively more abundant as surface temperature decreased (Table 3).

In addition, **Raman** data provided insights into the chemical allotropy (*i.e*., property of some chemical elements to exist in different structural forms) of some inorganic phases and their potential sources. For instance, the position of **native sulfur** peaks and their shifts relative to the RRUFF database allowed distinguishing the sulfur allotrope present in a sample. In the high-temperature samples MAT-78 or MP-87, the peaks of native sulfur were observed to be blueshifted (*i.e*., 231 and 479 cm^-1^) relative to the characteristic peaks in the RRUFF database (220 and 473 cm^-1^), as well as to slightly broaden and relatively intensity reduced (Fig. 5). These anomalies were related to the dominance of γ-sulfur, a yellow allotrope that forms by slow cooling of sulfur previously molten at temperatures above 150º [51]. In contrast, native sulfur peaks in low-temperature samples such as IF-20 or IF-49 fitted well with the characteristic sulfur peaks in the RRRUFF database that correspond to α-sulfur, the most common sulfur allotrope, whitish, that typically forms at low temperatures [51]. Other studies have associated the presence of poorly crystalline γ-sulfur with biological activity observing the predominance of this allotrope inside cells of γ-Proteobacteria (*Thiothrix*; [52]) or *Nitrospirae* (*Candidatus* *Magnetobacterium bavaricum*; [53]) in comparison to the mixture of the three allotropes (α-, β- and γ-S8) found outside the bacteria. The dominance of amorphous γ-sulfur observed in our high-temperature samples MAT-78 or MP-87 could be explained in some extent by contribution of biological activity.

Similarly, peculiarities in the Raman spectrum of **hematite** in IF-49 allowed discussing potential origins of the mineral. In addition to the hematite-characteristic peaks at 225, 295, 410, and 1320 cm^-1^, a broad band around 800 cm^-1^ was also observed in the Raman spectrum of IF-49 (Fig. 5). The amorphization suggested by this broad peak, not observed before, might be caused by inorganic hydrothermalism [54]. However, a similar broadening at 660 cm^-1^ in microstromatolites from a white smoker-type seafloor hydrothermal environment was associated by Kilias and coworkers [55] to the active participation of microbes in the mineral synthesis. Although the position of our peak was not exactly the same as that found by them, the amorphization could have been similarly caused. While our data do not allow us to conclusively discriminate between a hydrothermal or biological origin of the observed broad peak at 800 cm^-1^ in IF-49, biomediation influence cannot be completely excluded.

Other inorganic phases such as **anatase** were identified in some samples (AF-25, AF-90, IF-20, IF-49, and IF-74; Fig. 5), based on the detection of three characteristic peaks (395, 515 cm^-1^, and 635 cm^-1^) [54, 56].

Finally, Raman spectroscopy was also useful to detect a signal compatible with **carotenoid** in MAT-54 (Fig. 5), with three typical signals around 1000, 1150 and 1510 cm^-1^ resulting from C-CH_3_, C-C, and C=C stretching vibrations respectively [57].

**
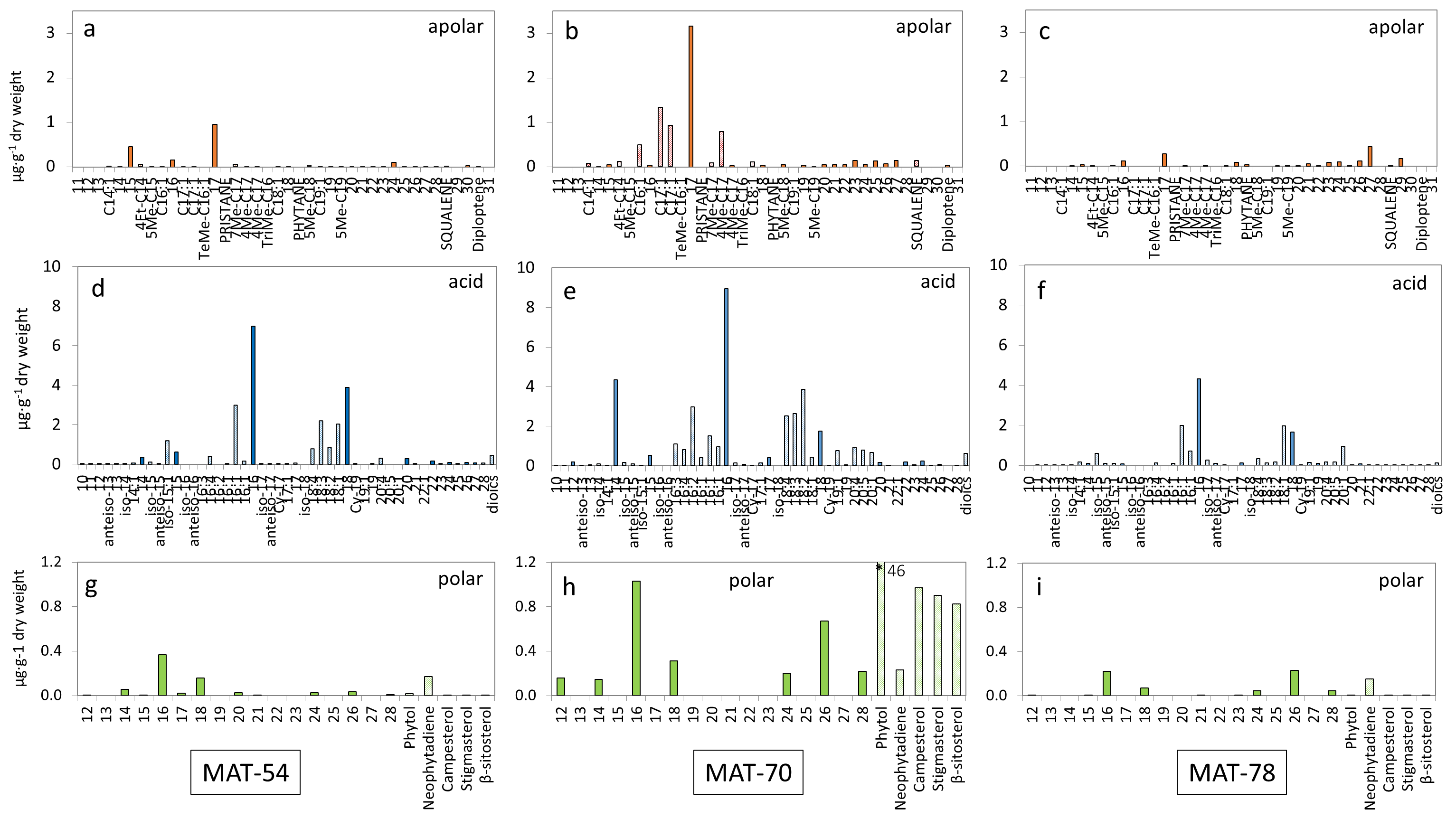
**

**Fig. S1**. Molecular distribution of hydrocarbons (apolar; a-c), fatty acids (acid, d-f), and alcohols (polar, g-i) in the three hot spring **biofilms** at 54ºC (MAT-54), 70ºC (MAT-70), and 78ºC (MAT-78). In the three polarity fractions, saturated straight-chain (*i.e.* *normal*) moieties (*n-*alkanes, *n-*fatty acids, and *n-*alkanols) are shown with dark colored bars, while other compounds with pale colored bars.


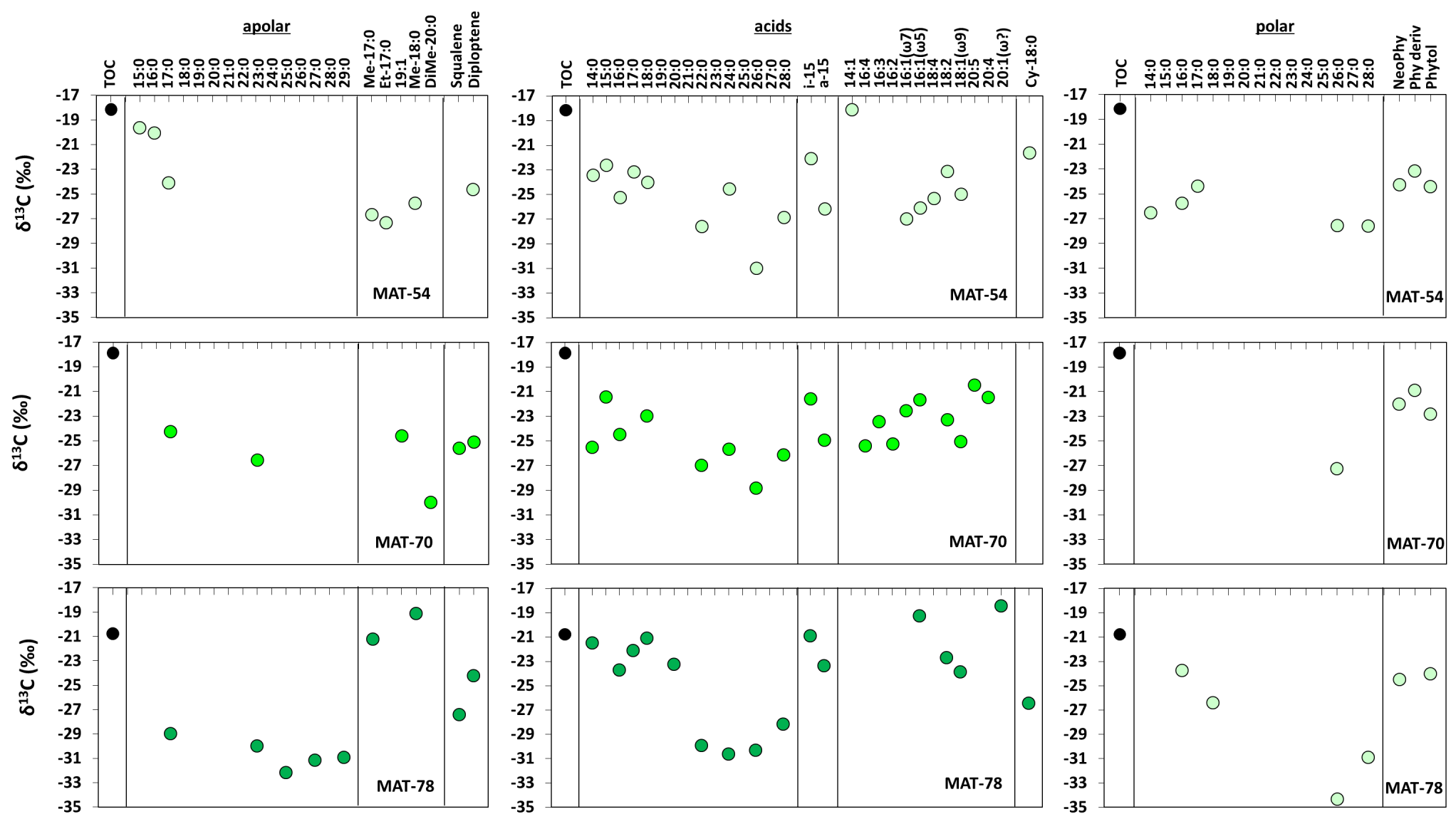


**Fig. S2**. Stable carbon isotopic composition of the bulk biomass (*i.e.*, TOC, black dots) and the three lipidic fractions (green dots) in the three hot spring **biofilms** (MAT-54, MAT-70, and MAT-78).

**
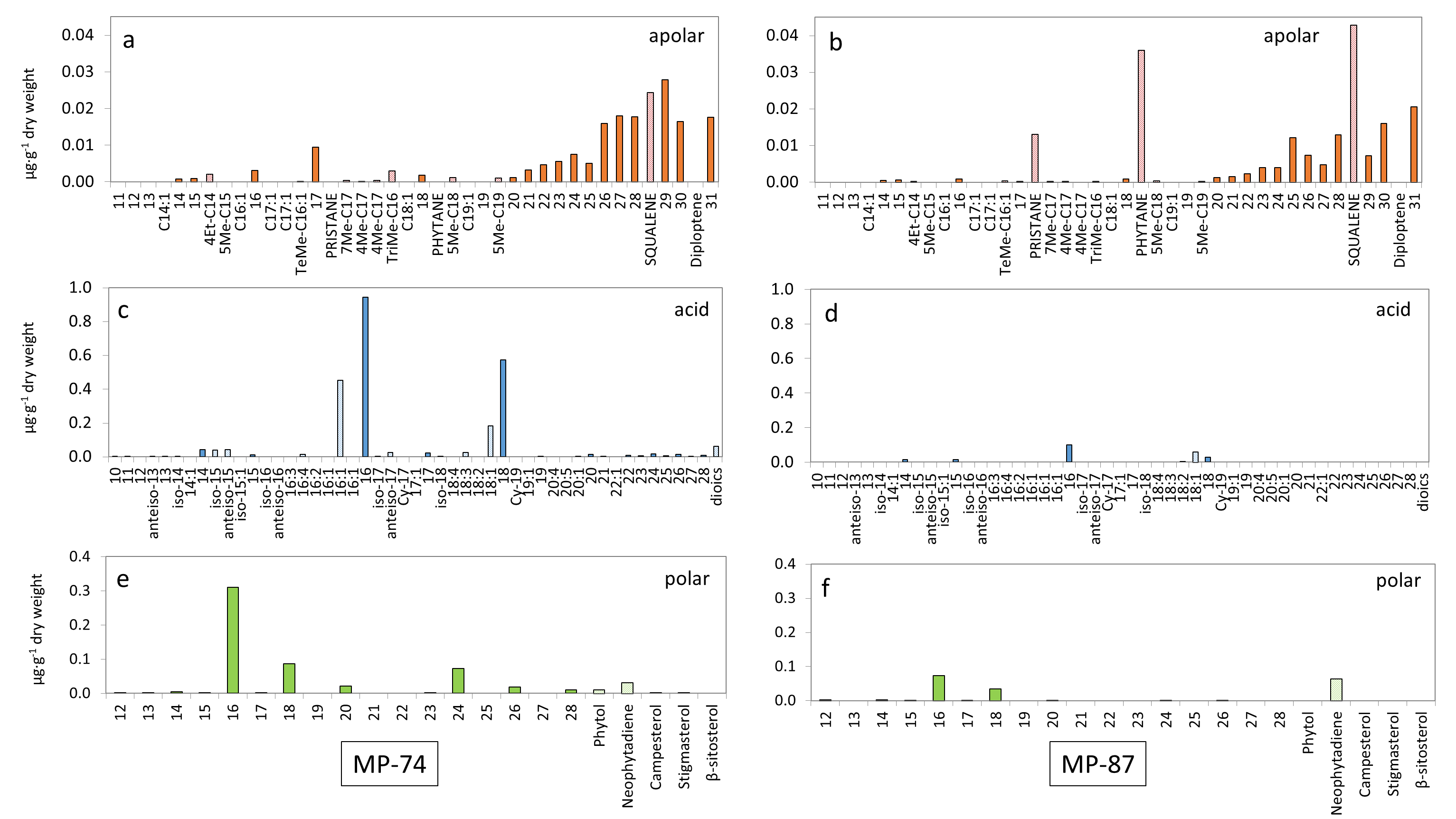
**

**Fig. S3**. Molecular distribution of hydrocarbons (apolar, a-b), fatty acids (acid, c-d), and alcohols (polar, e-f) in the two **mud pots** at 74ºC (MP-74) and 87ºC (MP-87). In the three polarity fractions, saturated straight-chain (*i.e.* *normal*) moieties (*n-*alkanes, *n-*fatty acids, and *n-*alkanols) are shown with dark colored bars, while other compounds with pale colored bars.

**
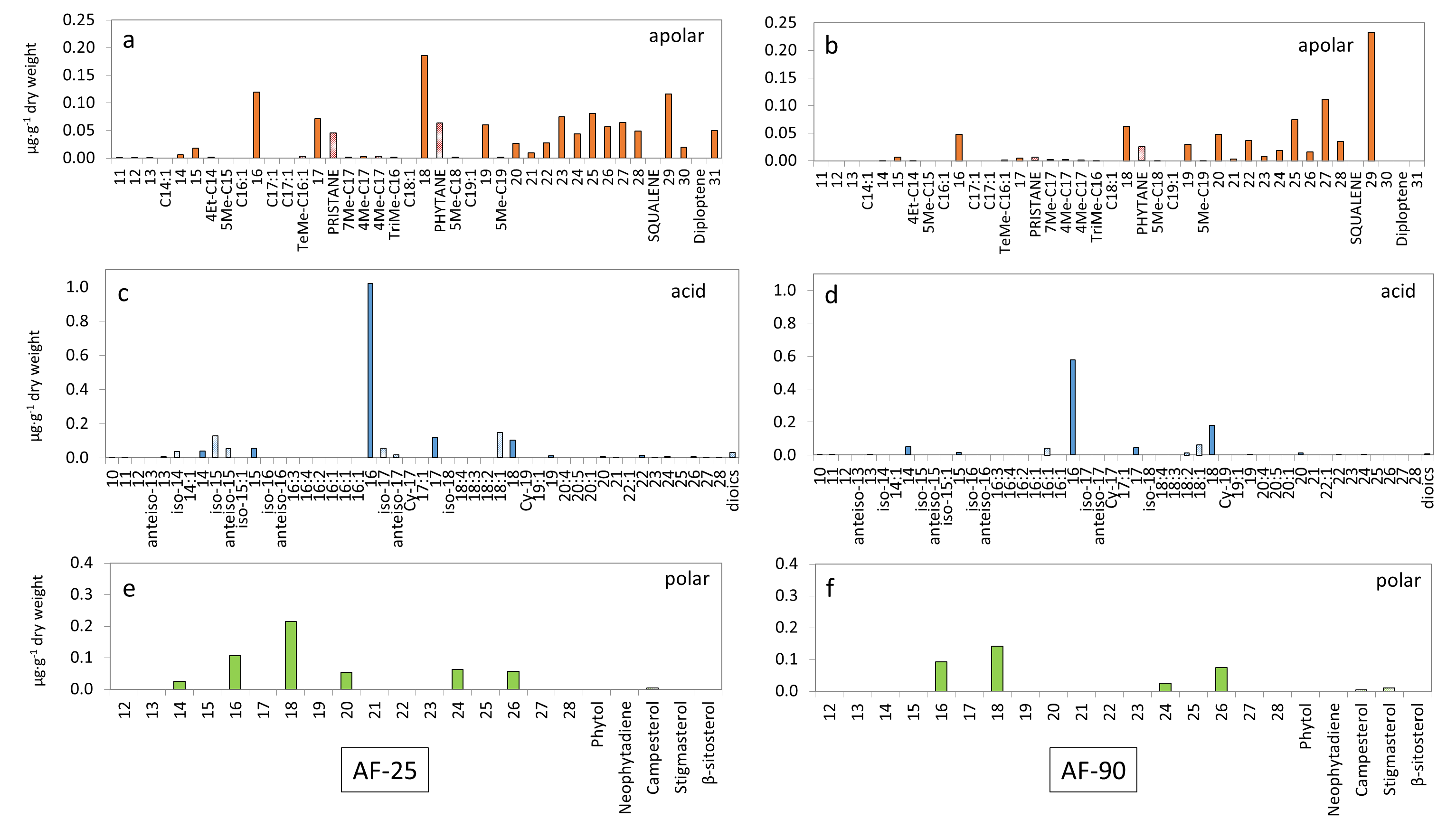
**

**Fig. S4**. Molecular distribution of hydrocarbons (apolar, a-b), fatty acids (acid, c-d), and alcohols (polar, e-f) in the two **active fumaroles** at 25ºC (AF-25) and 90ºC (AF-90). In the three polarity fractions, saturated straight-chain (*i.e.* *normal*) moieties (*n-*alkanes, *n-*fatty acids, and *n-*alkanols) are shown dark colored bars, while other compounds with pale colored bars.

**
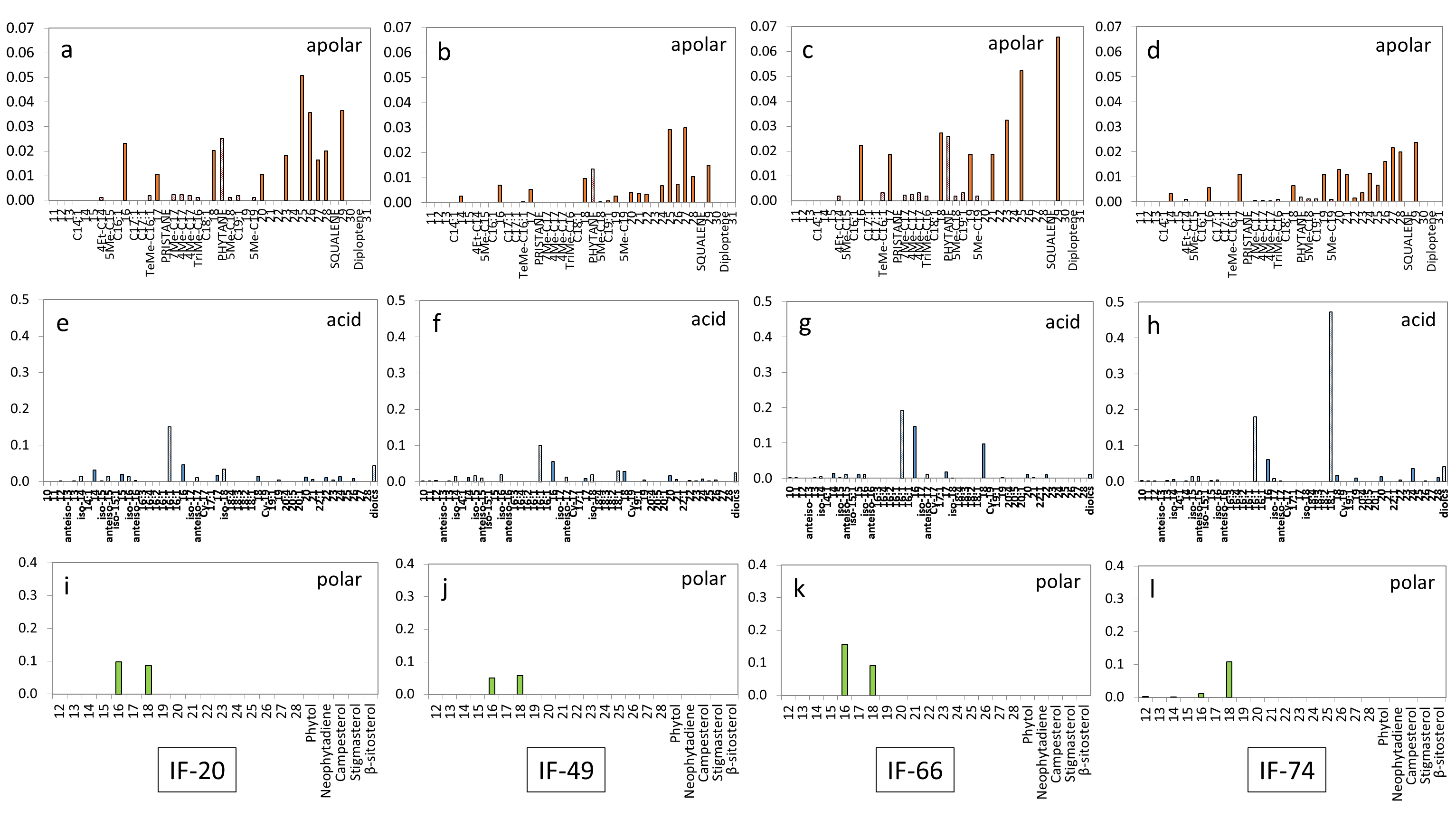
**

**Fig. S5**. Molecular distribution of hydrocarbons (apolar, a-d), fatty acids (acid, e-h), and alcohols (polar, i-l) in the four **inactive fumaroles** at 20ºC (IF-20), 49ºC (IF-49), 66ºC (IF-66) and 90ºC (IF-90). Please note that concentration units (µg·g^-1^ dw) are not shown in the Y axis due to space constrains. In the three polarity fractions, saturated straight-chain (*i.e.*, *normal*) moieties (*n-*alkanes, *n-*fatty acids, and *n-*alkanols) are shown dark colored bars, while other compounds with pale colored bars.

**
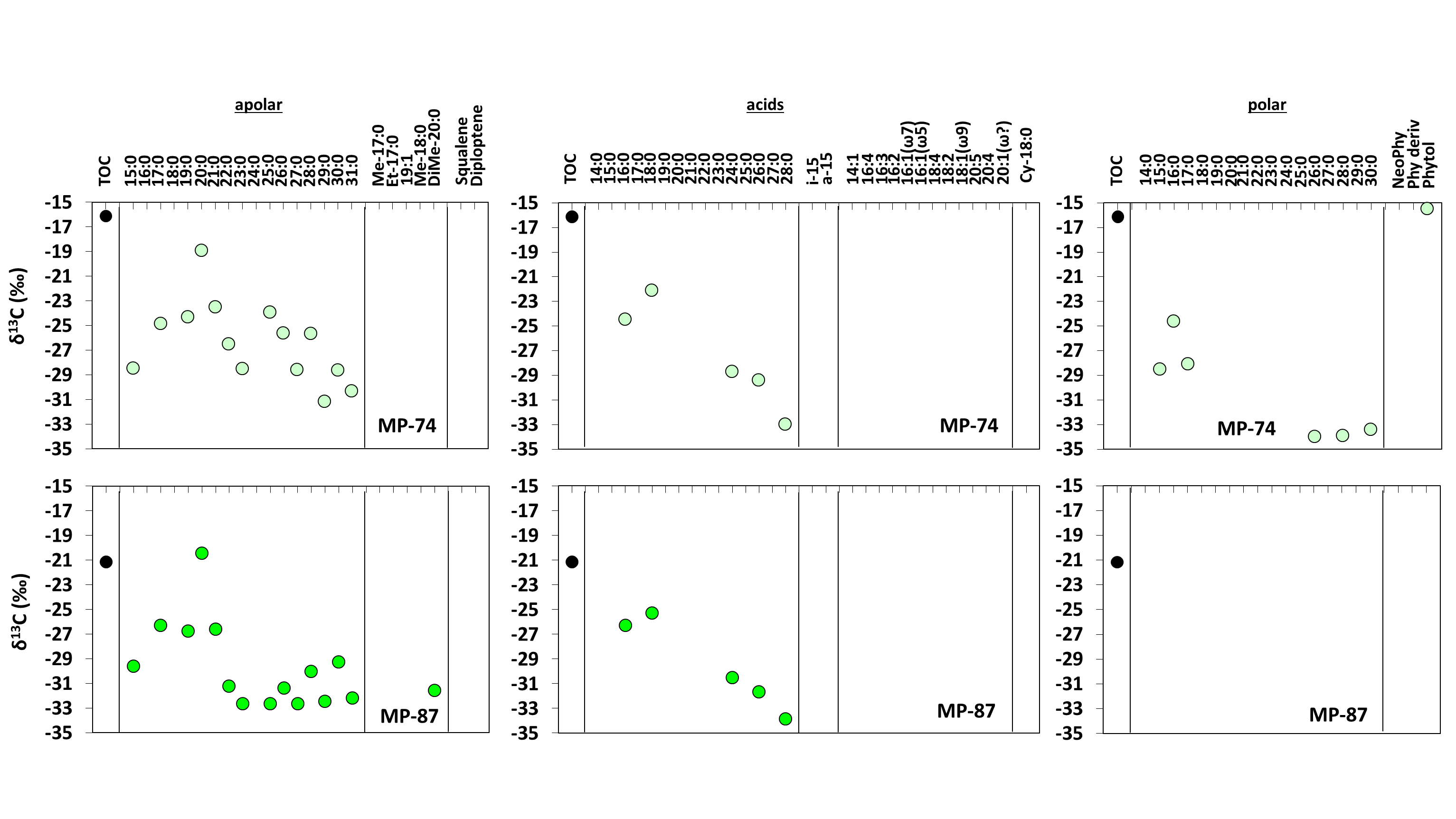
**

**Fig. S6**. Stable carbon isotopic composition of the bulk biomass (*i.e.*, TOC, black dots) and the three lipidic fractions (green dots) in the two **mud pots** (MP-74 and MP-87).


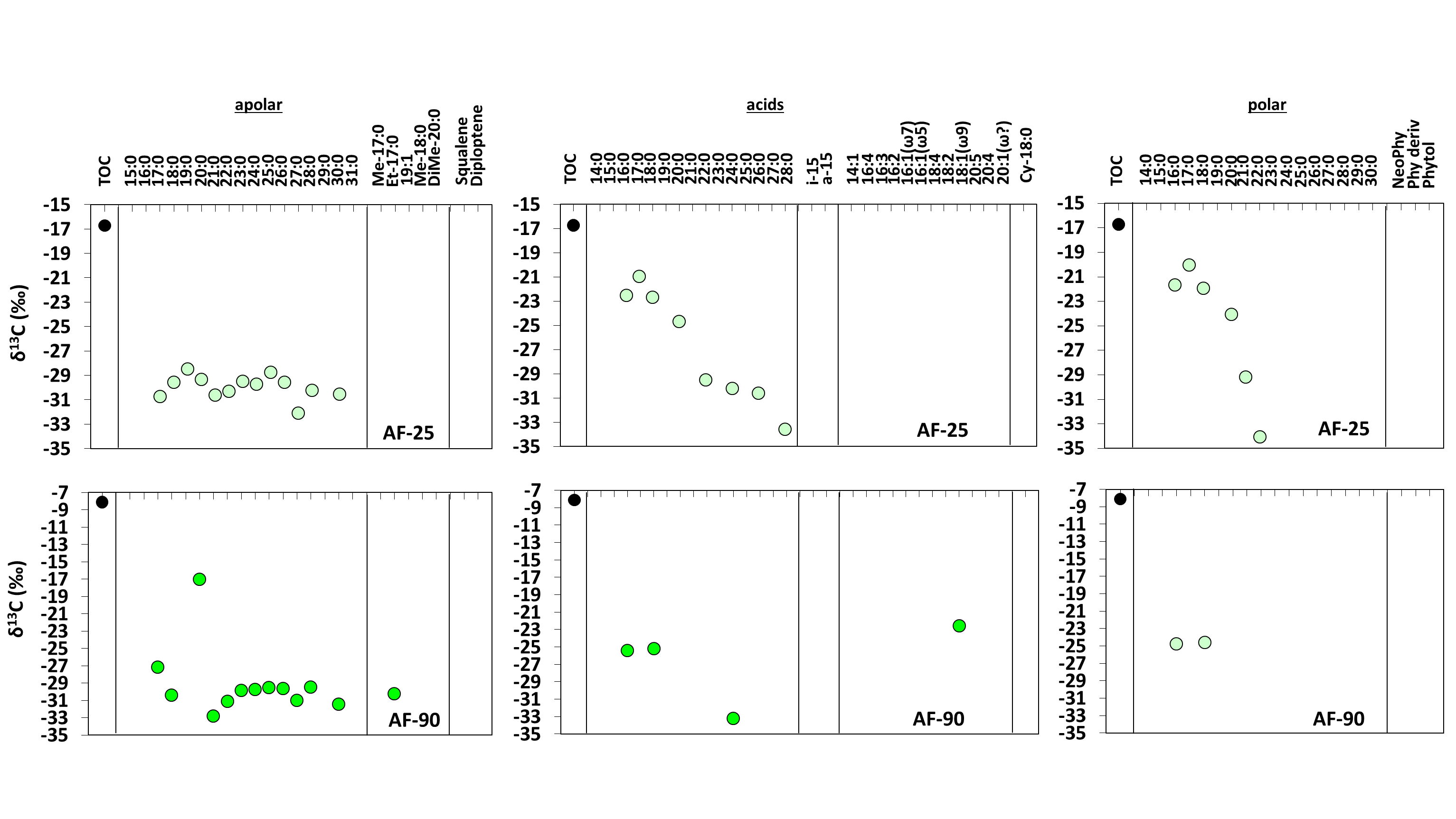


**Fig. S7**. Stable carbon isotopic composition of the bulk biomass (*i.e.*, TOC, black dots) and the three lipidic fractions (green dots) in **the active fumaroles** (AF-25 and AF-90).

**
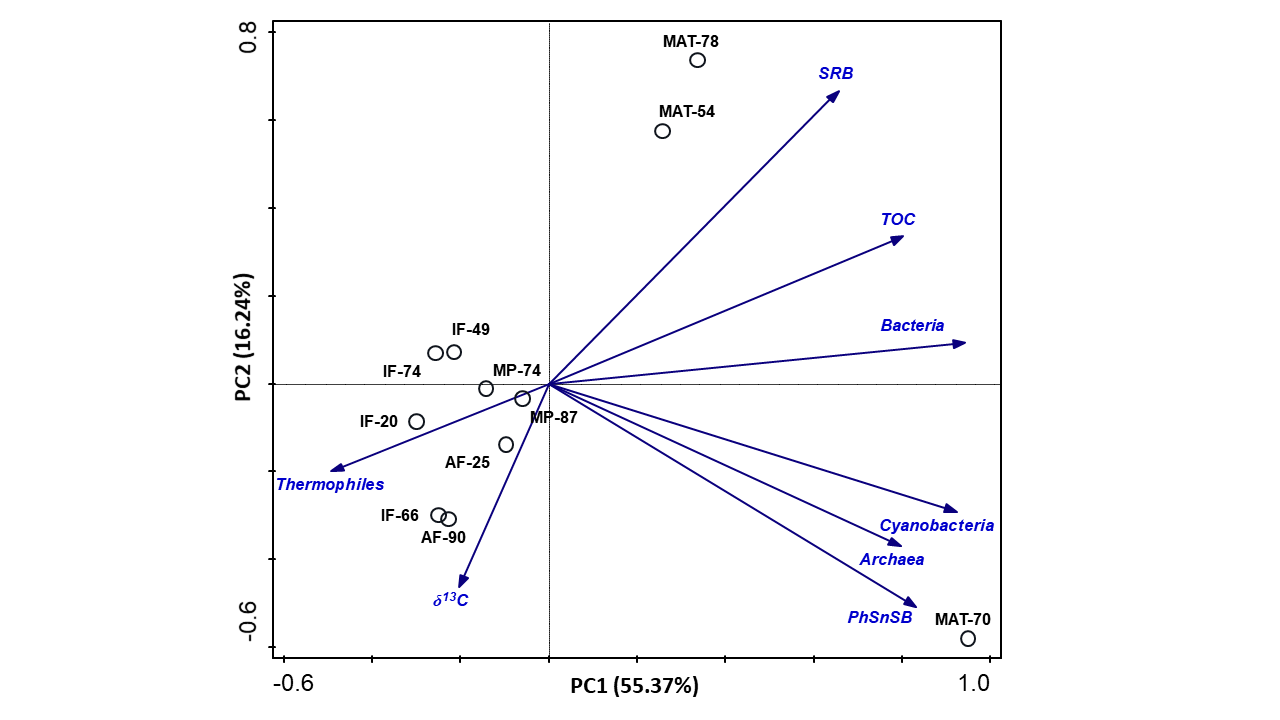
**

**Fig. S8**. Ordination plot of Principal Component Analysis (PCA) on the Icelandic hydrothermal samples. The samples names (black) are composed of initials describing the type of sample (MAT for biofilms, MP for mud pots, AF for active fumaroles, and IF for inactive fumaroles) and numbers indicating the temperature recorded *in situ* at the time of collection. Blue vectors represent the nine compositional variables tested; biomass content (*i.e*., TOC), stable carbon isotopic composition of biomass (δ^13^C), and individual lipid biomarkers of bacteria, cyanobacteria, PhSnSB, SRB, archaea, and thermophiles. Principal components PC1 and PC2 explained together 71.61% of the compositional variance.





**Fig. S9**. Example of Near Infrared spectra showing the presence of the main bands of Opal in samples IF-66 and IF-74: doublet at 1.4 and 1.46 (overtone of -OH stretch), 1.9 (–OH stretch, H-O-H bend), 2.21 (–OH stretch, Si–OH bend), 2.26 (–OH stretch, Si–OH bend).


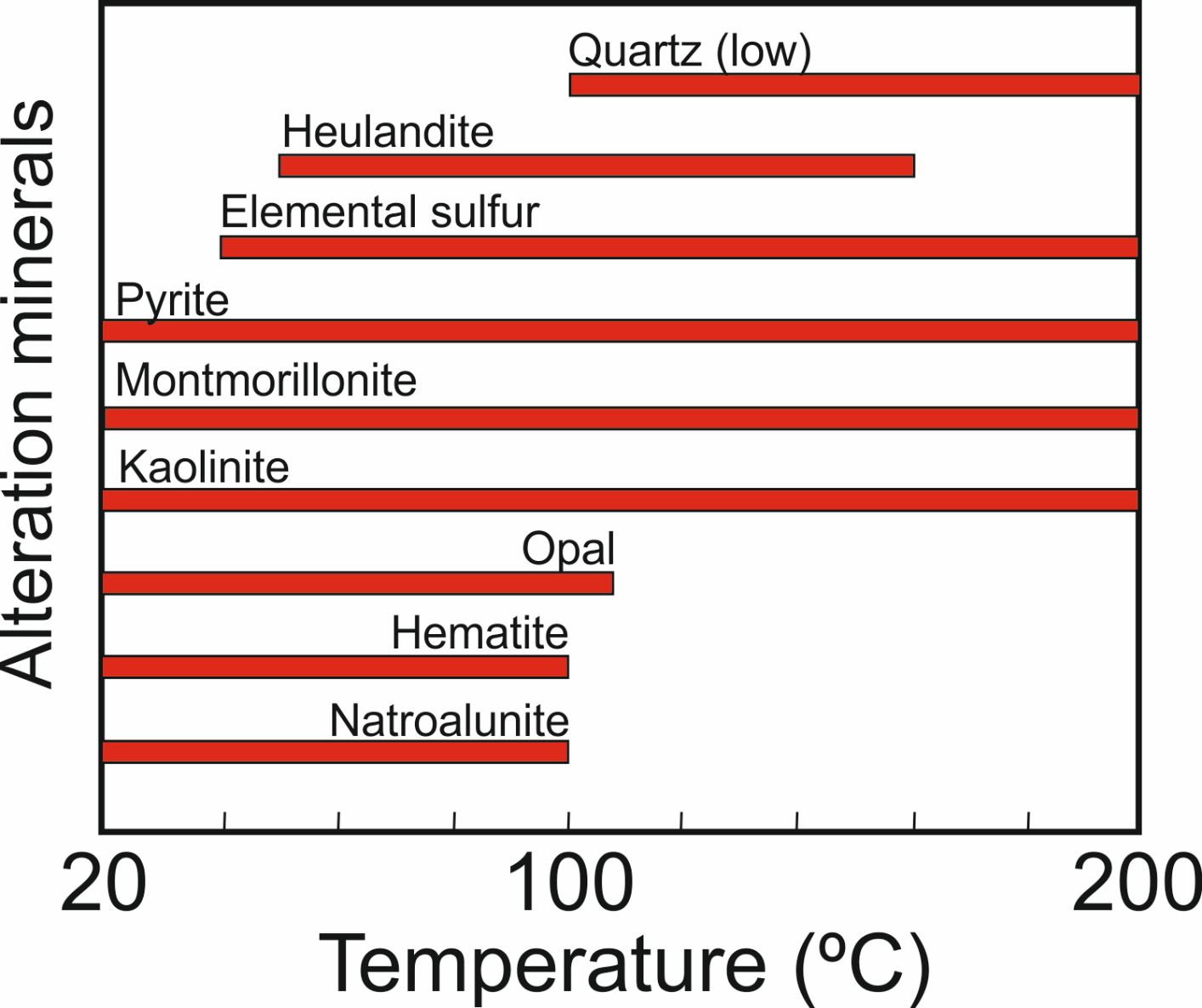


**Fig. S10**. Temperature stability constraints for the alteration minerals identified in the Icelandic hydrothermal scenarios.

**References**

1. White, D.C., Pinkart, H.C. & Ringelberg, A.B. Biomass measurements: biochemical approaches, in *Manual of Environmental Microbiology* (Eds. Hurst, C.J. *et al*.), 91–101 (ASM Press, Washington, DC, 1997).
2. Kaneda, T. *Iso*- and *anteiso*-fatty acids in bacteria: biosynthesis, function and taxonomic significance. *Microbiol. Rev.* **55**, 288–302 (1991).
3. Zelles, L. Phospholipid fatty acid profiles in selected members of soil microbial communities. *Chemosphere* **35**, 275–294 (1997).
4. Tornabene, T.G., Langworthy, T.A., Holzer, G. & Oro, J. Squalenes, phytanes and other isoprenoids as major neutral lipids of methanogenic and thermoacidophilic archaebacteria. *J. Mol. Evol.* **13**, 73–83 (1979).
5. Stiehl, T., Rullkötter, J. & Nissenbaum, A. Molecular and isotopic characterization of lipids in cultured halophilic microorganisms from the Dead Sea and comparison with the sediment record of this hypersaline lake. *Org. Geochem.* **36**, 1242–1251 (2005).
6. Gelpi, E., Scheider, H., Mann, J. & Oro, J. Hydrocarbons of geochemical significance in microscopic algae. *Phytochemistry* **9**, 603–612 (1970).
7. Shiea, J., Brassell, S.C. & Ward, D.M. Mid-chain branched mono- and dimethyl alkanes in hot spring cyanobacterial mats: A direct biogenic source for branched alkanes in ancient sediments. *Org. Geochem.* **15**, 223–231 (1990).
8. Kenig, F. *et al*. Structure and distribution of branched aliphatic alkanes with quaternary carbon atoms in Cenomanian and Turonian black shales of Pasquia Hills (Saskatchewan, Canada). *Org. Geochem.* **36**, 117–138 (2005).
9. Allen, M.A., Neilan, B.A., Burns, B.P., Jahnke, L.L. & Summons, R.E. Lipid biomarkers in Hamelin Pool microbial mats and stromatolites. *Org. Geochem.* **41**, 1207–1218 (2010).
10. Sakata, S. *et al*. Carbon isotopic fractionation associated with lipid biosynthesis by a cyanobacterium: relevance for interpretation of biomarker records. *Geochim. Cosmochim. Ac.* **61**, 5379–5389 (1997).
11. Ahlgren, G., Gustafsson, I.-B. & Boberg, M. Fatty acid content and chemical composition of freshwater microalgae. *J. Phicol.* **28**, 37–50 (1992).
12. Pagès, A. *et al*. Lipid biomarker and isotopic study of community distribution and biomarker preservation in a laminated microbial mat from shark bay, Western Australia. *Microb. Ecol*. **70**, 459–472 (2015).
13. Grossi, V. *et al*. Biotransformation pathways of phytol in recent anoxic sediments. *Org. Geochem.* **29**, 845–861 (1998).
14. Dowling, N.J.E., Widdel, F. & White, D.C. Phospholipid ester-linked fatty acid biomarkers of acetate-oxidizing sulfatereducing bacteria and other sulfide-forming bacteria. *J. Gen. Microbiol.* **132**, 1815–1825 (1986).
15. Londry, K.L. & Des Marais, D.J. Stable carbon isotope fractionation by sulfate-reducing bacteria. Applied and *Environ. Microbiol.* **69**, 2942–2949 (2004).
16. Konneke, M. & Widdel, F. Effect of growth temperature on cellular fatty acids in sulphate-reducing bacteria. *Environ. Microbiol.* **5**, 1063–1070 (2003).
17. Brocks, J.J., & Summons, R.E. Sedimentary hydrocarbons, biomarkers for early life, in *Biogeochemistry: Treatise on Geochemistry*, Vol. 8 (Eds. Schlesinger, W.H) 63–115 (Oxford: Elsevier Pergamon, 2003).
18. Didyk B.M., Simoneit B.R.T., Brassell S.C. & Eglinton G. Organic geochemical indicators of palaeoenvironmental conditions of sedimentation. *Nature* **272**, 216–222 (1978).
19. Fang, J. *et al*. Biomarker analysis of microbial diversity in sediments of a saline groundwater seep of Salt Basin, Nebraska. *Org. Geochem.* **37**, 912-931 (2006).
20. Carballeira, N.M. *et al*. Unusual fatty acid compositions of the hyperthermophilic archaeon *Pyrococcus furiosus* and the bacterium *Thermotoga maritima*. *J. Bacteriol.* **179**, 2766–2768 (1997).
21. Hefter, J. *et al*. Biomarker indications for microbial contribution to Recent and Late Jurassic carbonate deposits. *Facies* **29**, 93–105 (1993).
22. Chen, L. *et al*. Molecular records of microbialites following the end-Permian mass extinction in Chongyang, Hubei Province, South China. *Palaeogeogr. Palaeocl.* **308**, 151–159 (2011).
23. Killops, S.D. & Killops, V. J. *Introduction to Organic Geochemistry.* Blackwell Publishing, Oxford (2005).
24. Summons, R.E. *et al*. Lipid biomarkers in ooids from different locations and ages: evidence for a common bacterial flora. *Geobiology* **11**, 420-436 (2013).
25. Schinteie, R. & Brocks, J.J. Paleoecology of Neoproterozoic hypersaline environments: biomarker evidence for haloarchaea, methanogens, and cyanobacteria. *Geobiology* **15**, 641-663 (2017).
26. Hayes, J.M. *Fractionation of the isotopes of carbon and hydrogen in biosynthetic processes*, National Meeting of the Geological Society of America, Boston, MA, 31 pp (2001).
27. Preuss, A., Schauder, R. & Fuchs, G. Carbon isotope fractionation by autotrophic bacteria with three different CO_2_ fixation pathways. *Z. Naturforsch Teil C* **44**, 397–402 (1989).
28. van der Meer, M.T.J. *et al*. Biosynthetic controls on the ^13^C contents of organic components in the photoautotrophic bacterium Chloroflexus aurantiacus. *J. Biol. Chem.* **276**, 10971–10976 (2001).
29. Hügler, M. & Sievert, S.M. Beyond the Calvin Cycle: autotrophic carbon fixation in the ocean. *Annu. Rev. Mar. Sci.* **3**, 261–289 (2011).
30. Robinson, N. & Eglinton, G. Lipid chemistry of Icelandic hot spring microbial mats. *Org. Geochem.* **15**, 291–298 (1990).
31. Castenholz, R.W. The thermophilic cyanophytes of Iceland and the upper temperature limit. *J. Phycol.* **5**, 360–368 (1969).
32. Ward, D.M., Ferris, M.J., Nold, S.C. & Bateson, M.M. A natural view of microbial biodiversity within hot spring cyanobacterial mat communities. *Microbiol. Mol. Biol. Rev.* **62**, 1353–1370 (1998).
33. Jørgensen, B.B. & Nelson, D.C. Bacterial zonation, photosynthesis, and spectral light distribution in hot spring microbial mats of Iceland. *Microb. Ecol.* **16**, 133–147 (1988).
34. Van der Meer, M.T.J, Lammerts, L., Skirnisdottir, S., Sinninghe Damsté, J.S. & Schouten, S. distribution and isotopic composition of bacterial lipid biomarkers in microbial mats from a sulfidic Icelandic hot spring. *Org. Geochem.* **39**, 1015–1019 (2008).
35. Bodelier, P.L.E. *et al*. A reanalysis of phospholipid fatty acids as ecological biomarkers for methanotrophic bacteria. *ISME J.* **3**, 606–617 (2009).
36. Dijkman, N.A., Boschker, H.T.S., Stal, L.J. & Kromkamp, J.C. Composition and heterogeneity of the microbial community in a coastal microbial mat as revealed by the analysis of pigments and phospholipid-derived fatty acids. *J. Sea Res.* **63**, 62–79 (2010).
37. Volkman, J.K. A review of sterol markers for marine and terrigenous organic matter. *Org. Geochem.* **9**, 83–99 (1986).
38. Eglinton, G. & Hamilton, R.J. Leaf epicuticular waxes. *Science* **156**, 1322–1335 (1967).
39. Van der Meer, M.T.J., Schouten, S., de Leeuw, J.W. & Ward, D.W. Autotrophy of green non-sulfur bacteria in hot spring microbial mats: biological explanations for isotopically heavy organic carbon in the geological record. *Environ. Microbiol.* **2**, 428–435 (2000).
40. Pierson, B.K. & Castenholz, R.W. Studies of pigments and growth in Chloroflexus aurantiacus, a phototrophic filamentous bacterium. *Arch. Microbiol.* **100**, 283–305 (1992).
41. Ward, D.M., Tayne, T.A., Anderson, K.L. & Bateson, M.M. Community structure, and interactions among community members in hot spring cyanobacterial mats. *Symp. Soc. Gen. Microbiol.* **41**, 179–210 (1987).
42. Vestal, J.R. & White, D.C. Lipid analysis in microbial ecology. *Bioscience* **39**, 535–541 (1989).
43. Langworthy, T.A., Holzer, G., Zeikus, J.G. & Tornabene, T.G. *Iso*- and *anteiso*-branched glycerol diethers of the thermophilic anaerobe *Thermodesulfotobacterium commune*. *Syst. Appl. Micrbiol.* **4**, 1–17 (1983).
44. Tobler, D.J. & Benning, L.G. Bacterial diversity in five Icelandic geothermal waters: temperature and sinter growth rate effects. *Extremophiles* **15**, 473–485 (2011).
45. Cirés, S., Casero M.C. & Quesada A. Toxicity at the edge of life: a review of cyanobacterial toxins from extreme environments. *Mar. Drugs* **15**, doi:10.3390/md15070233 (2017).
46. Skirnisdottir, S. *et al*. Influence of sulfide and temperature on species composition and community structure of hot spring microbial mats. *Appl. Environ. Microb.* **66**, 2835–2841 (2000).
47. Lynne, B.Y. Impact of three common post-depositional environmental settings on siliceous sinter diagenesis: an eight year experiment. J. Volcanol. Geotherm. Res. 292, 84–101 (2015).
48. Fournier, R. O. Geochemistry and dynamics of the Yellowstone National Park Hydrothermal System. Annu. Rev. Earth Planet. Sci. 177, 13–53 (1989).
49. Lezcano, M.A. et al. Biomarker profiling of microbial mats in the geothermal band of Cerro Caliente, Deception Island (Antarctica): life at the edge of heat and cold. Astrobiology 19, 1490-1504 (2019).
50. Sánchez-García et al. Microbial biomarker transition in high-altitude sinter mounds from El Tatio (Chile) through different stages of hydrothermal activity. *Front. Microbiol*. ﻿**9**, 3350 (2019).
51. Steudel, R., ed. (2004). Elemental sulfur and sulfur-rich compounds I (Topics in current chemistry). Springer. ISBN 3-540-40191-1.
52. Nims, C., Cron, B., Wetherington, M., Macalady, J., Cosmidis, J. Low frequency Raman Spectroscopy for micron-scale and in vivo characterization of elemental sulfur in microbial samples. *Sci Rep* **9**, 7971 (2019).
53. Eder, S.H., Gigler, A.M., Hanzlik, M. & Winklhofer, M. Sub-micrometer-scale mapping of magnetite crystals and sulfur globules in magnetotactic bacteria using confocal Raman micro-spectrometry. *PLoS One*. **9**, e107356 (2014).
54. Ruiz‐Galende *et al*. Study of a terrestrial Martian analogue: Geochemical characterization of the Meñakoz outcrops (Biscay, Spain). *J Raman Spectrosc*. 1– 10, doi 10.1002/jrs.5565 (2019).
55. Kilias, S., Chatzitheodoridis, E. & Lyon, I. Molecular, chemical and morphological evidence for hematite biogenicity at the Quaternary Cape Vani Mn-(Ba-Fe) deposit, Milos, Greece. *Bulletin of the Geological Society of Greece* **47**, 834-842 (2013).
56. Glamoclija, M. *et al*. Association of anatase (TiO_2_) and microbes: Unusual fossilization effect or a potential biosignature? *The ICDP-USGS Deep Drilling Project in the Chesapeake Bay impact structure*: Results from the Eyreville Core Holes, Gregory S. Gohn, Christian Koeberl, Kenneth G. Miller, Wolf Uwe Reimold (2009).
57. Gall, A., Pascal, A.A. & Robert, B. Vibrational techniques applied to photosynthesis: Resonance Raman and fluorescence line-narrowing. BBA-Bioenergetics 1847, 12-18 (2015).
